# Goldilocks-Inspired Design of Mid-Size Macrocycles for Selective Targeting of Human Melanocortin Receptors

**DOI:** 10.1021/acs.jmedchem.6c01074

**Published:** 2026-06-24

**Authors:** Francesco Merlino, Li Jia, Ida Boccino, Alfonso Carotenuto, Francesco Tammaro, Federica Santoro, Philip E. Thompson, Paolo Grieco

**Affiliations:** † Department of Pharmacy, 9307 University of Naples Federico II, via Domenico Montesano 49 80131, Naples, Italy; ‡ Medicinal Chemistry. Monash Institute of Pharmaceutical Sciences, Faculty of Pharmacy and Pharmaceutical Sciences, 2541 Monash University Parkville Campus, 381 Royal Parade, Parkville VIC, Melbourne 3052, Australia

## Abstract

The recent clinical success of macrocyclic peptides, such as bremelanotide and setmelanotide, highlights the potential of this modality as a “Goldilocks” chemical class, effectively balancing the properties of small molecules and biologics while optimizing their affinity, stability, and pharmacokinetics. Despite their potential, achieving selective MCR targeting with optimal pharmacological profiles remains challenging. Motivated by these considerations, we designed and synthesized a series of 14 macrocyclic peptide analogs (19–23-membered rings) based on **MT-II**, **SHU-9119**, and **PG-901** scaffolds. Their pharmacological activities were assessed using human MC1R, MC3R, MC4R, and MC5R. The novel macrocyclic compounds **FM648** and **FM636** emerged as potent and selective *h*MC4R antagonists. Additionally, **FM635** exhibited interesting agonist activity with remarkable selectivity for the same receptor. This study underscores the potential of tailored midsized macrocycles as selective MCR ligands and provides new insights into the structure–activity relationships, thereby guiding the development of therapeutic candidates for metabolic and inflammatory diseases.

## Graphical abstract

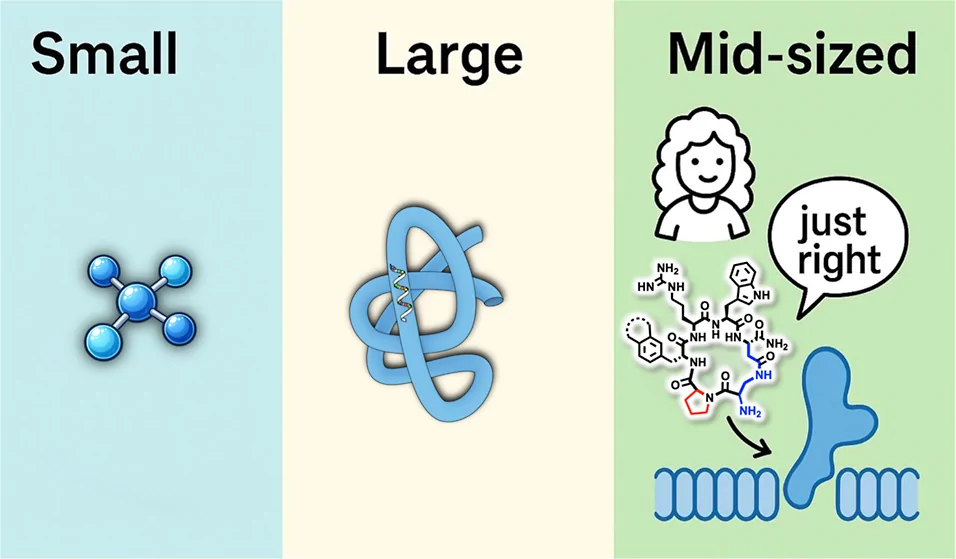

## Introduction

Melanocortins are a group of small peptides that include adrenocorticotropic hormone (ACTH) and melanocyte-stimulating hormones α-MSH, β-MSH, and γ-MSH. They originate from a common source, pro-opiomelanocortin (POMC), and act via melanocortin receptors (MC1R-MC5R). These proteins are produced through specific processes in different tissues and help control functions such as skin color, hormone production, energy balance, and eating habits.^1–4^

The five melanocortin receptors, MC1R-MC5R, are part of the class A (rhodopsin-like) family of G protein-coupled receptors (GPCR). These receptors are found in different tissues and have different roles in the body. They are connected to adenyl cyclase (AC) and mainly activate the cAMP-dependent pathways. In addition to melanocortin proteins, other natural proteins, such as agouti signaling protein (ASIP), agouti-related protein (AgRP), and β-defensin 3, bind to these receptors. Because the melanocortin system is involved in many body functions, it is an important target for treating disorders such as obesity, muscle wasting, and inflammatory diseases.^5–9^

Targeting melanocortin receptors (MCRs) is a promising approach for treating metabolic disorders and chronic inflammation; however, developing drugs for this purpose is difficult because of selectivity and safety concerns. Recently, a drug called [Nle,^4^D­(2′)­Phe]­α-MSH (afamelanotide) was approved in Europe and the US for the treatment of erythropoietic protoporphyria (EP). Other drugs, such as bremelanotide and setmelanotide, have also been approved.^10,11^ Therefore, developing new drugs that target MCRs to treat obesity and its related conditions is crucial. Although some efforts have focused on repurposing known drugs, the design of drugs that specifically target these receptors remains complex.^12–14^ However, the approval of these two new drugs has led to the development of strong macrocyclic peptide drugs that are effective against MCR. These macrocyclic peptides have unique properties that differentiate them from other drugs, offering both advantages and challenges in their application. Similar to small molecules, they can be readily fabricated and improved using traditional chemical methods. This means that their properties, such as binding, stability, and solubility, can be adjusted for specific applications.^15,16^

Macrocyclic peptides are often referred to as the “Goldilocks” of drugs because they are the “just right” size, combining the best features of small molecules and biologics. Their ring shape allows them to cover a larger area, which can help them interfere with protein interactions more effectively than traditional linear peptides. These unique features offer good opportunities to address these challenges. Inspired by these insights and previous research on macrocyclic structures, we aimed to better understand the design of macrocyclic compounds that specifically target MCs.^17–20^

We synthesized 14 macrocyclic peptide analogs inspired by the macrocyclic frameworks of **MT-II**, **SHU-9119**, and **PG-901** with varying ring sizes (19–21 members) and evaluated their pharmacological properties on *h*MC1, *h*MC3, *h*MC4, and *h*MC5 receptors (Figure 1).^21,22^

**Figure 1:**
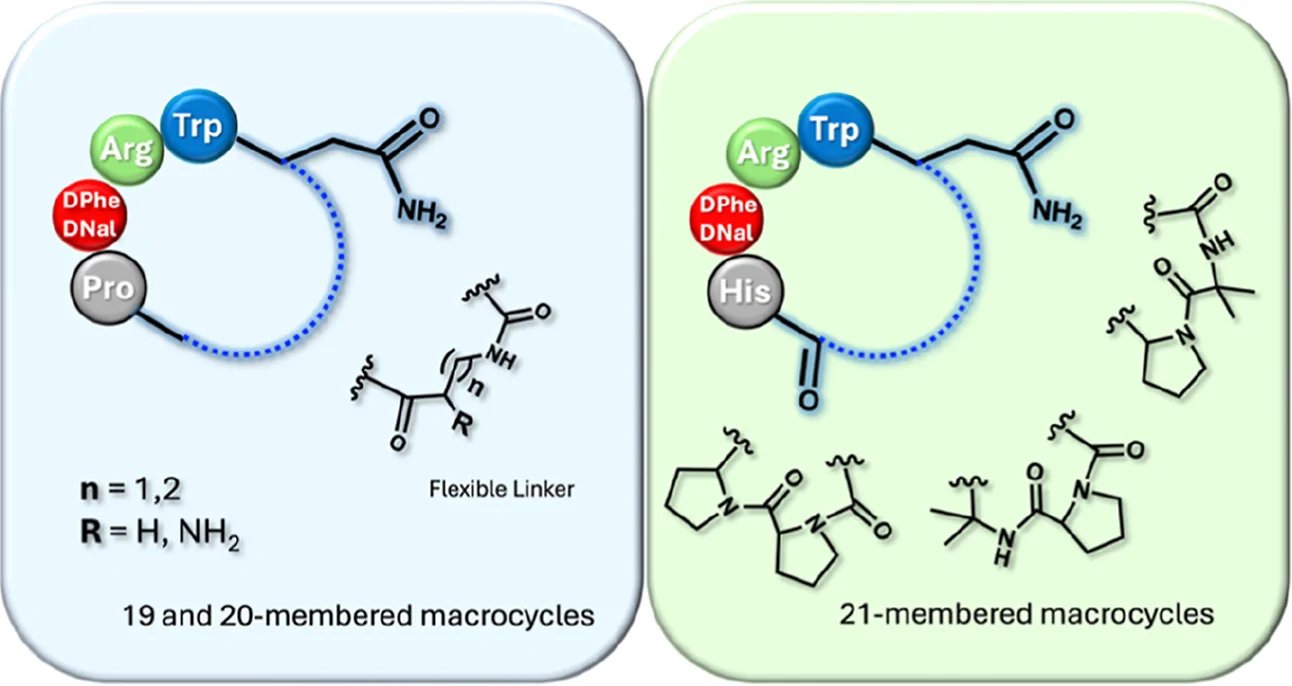
General scheme of synthesized macrocycles.

This study was based on the hypothesis that tailored macrocyclic designs can achieve an optimal balance between the affinity and receptor selectivity of ligands.

## Results and Discussion

### Design Strategy and Peptide Synthesis

To examine the effects of ring-contracted analogs of other significant **MT-II** derivatives, this study focused on **MT-II** and three of its analogs: **SHU-9119**, **Pro** ^6^ **-MT-II**, and **PG-901**.^23^ Notably, **Pro** ^6^ **-MT-II** and **PG-901** are characterized by the absence of the His^6^ residue, which is replaced by conformationally restricted proline.^24,25^ Furthermore, **PG-901** and **SHU-9119** contain a D(2′)­Nal residue that imparts antagonistic activity to these molecules at MC3R and MC4R. The proposed structural modifications introduce rigidity and induce conformational changes that may influence receptor binding and activity.

Grieco *et al.* previously demonstrated the existence of two turn motifs in **MT-II** and **SHU-9119** derivatives. These turns most closely resembled type I (residue 4 to 7) and type II (residue 5 to 8) β-turns.^26^ Substituting DPhe with D(2′)­Nal resulted in receptor antagonism and enhanced subtype selectivity, particularly for MC3R and MC4R. The theoretical rationale for this antagonism is that bulky groups, such as D(2′)­Nal, at the DPhe position may hinder the rotation of key receptor residues, such as Trp^258^ and Leu^133^, in MC4R, thereby stabilizing the receptor in an inactive state and promoting antagonism.^27^ D(2′)­Nal also enhances hydrophobic interactions and modifies the side-chain orientation, contributing to the observed antagonism. These structural modifications significantly reduced the agonistic potency of **PG-901** but markedly improved its receptor selectivity compared to that of **MT-II**. Bednarek *et al.* further explored the replacement of the core sequences His, Phe, Trp and Arg with Proline. Their findings indicated that substituting specific residues with proline often disrupts activity, underscoring the importance of certain side chains in receptor interaction. Interestingly, substituting His^6^ with Pro^6^ did not significantly affect the activity of MC4R, suggesting that the imidazole group of His is not essential for receptor binding.^28^ Recently, it has been demonstrated that His and Pro are interchangeable. When Pro^6^ replaces His^6^, the peptide maintains a similar backbone orientation. The main chain’s orientation indeed seems to be more essential than the presence of the His side chain.^28^ These findings underscore the importance of strategically modifying peptide design to achieve receptor-specific activity and selectivity.

In our design strategy, we investigated the impact of alternative conformational constraints on the biological activities of peptide analogs of **MT-II**, **SHU9119**, **Pro** ^6^ **-MT-II**, and **PG-901**.^29,30^ These analogs feature a modified structure in which the traditional side-chain bridge is replaced with shorter bonds connecting the C-terminal amino acid side chain to the *N*-terminal residue. It is well-known that the three-dimensional conformation of cyclic peptides is closely related to their molecular properties. As the ring dimensions vary, the structural elements can interact with the target, that is, with various receptor subtypes. Thus, variation of the ring size enables exploration of receptor selectivity within a given class of macrocyclic peptides.^31^ Solid-phase macrocyclization was achieved using two distinct strategies utilizing a peptide chain immobilized via the β-carboxamide of allyl aspartic acid, as shown in Scheme 1. For peptides containing a head-to-side chain cyclization, the lactam bridge was introduced according to the procedure reported in panel A (representative synthesis of **FM636**). For peptides containing a head-to-tail cyclization, the procedure is reported in panel B (representative synthesis of **FM641**). Figures 2 and 3 show the macrocycles with 19- and 20-membered rings and 21-membered rings, respectively. Analytical data of synthesized peptides are reported in Table S1 and Figures S1–S15 of the Supporting Information.

**Scheme 1:**
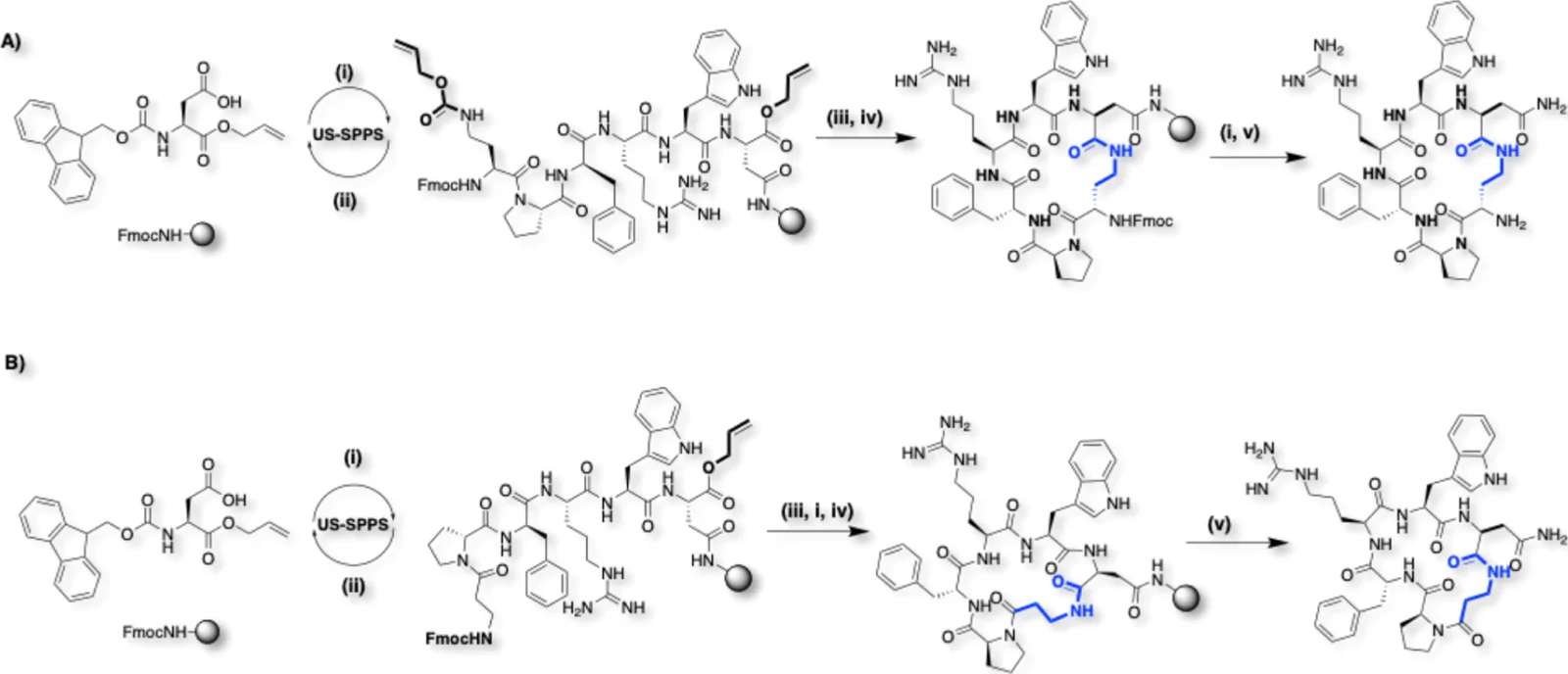
Synthetic Route for Macrocycles Peptides^a^

a (A) Procedure for the synthesis of peptides with head-to-side-chain cyclization. (B) Procedure for the synthesis of peptides with head-to-tail cyclization. (i) 20% piperidine in DMF (0.5 + 1 min) under ultrasound (US) irradiation; (ii) Fmoc-AA-OH (2 equiv), COMU (2 equiv), OXYMA (2 equiv), DIPEA (4 equiv) in DMF under US irradiation; (iii) Pd­(PPh_3_)_4_ (0.15 equiv), NDMBA (3 equiv), DCM/DMF 2:1 (v/v), 2 × 60 min; (iv) PyAOP (2 equiv), DIPEA (4 equiv) in DMF, rt, 16 h; (v) TFA, TIS, H_2_O (95:2.5:2.5, v/v/v).

**Figure 2:**
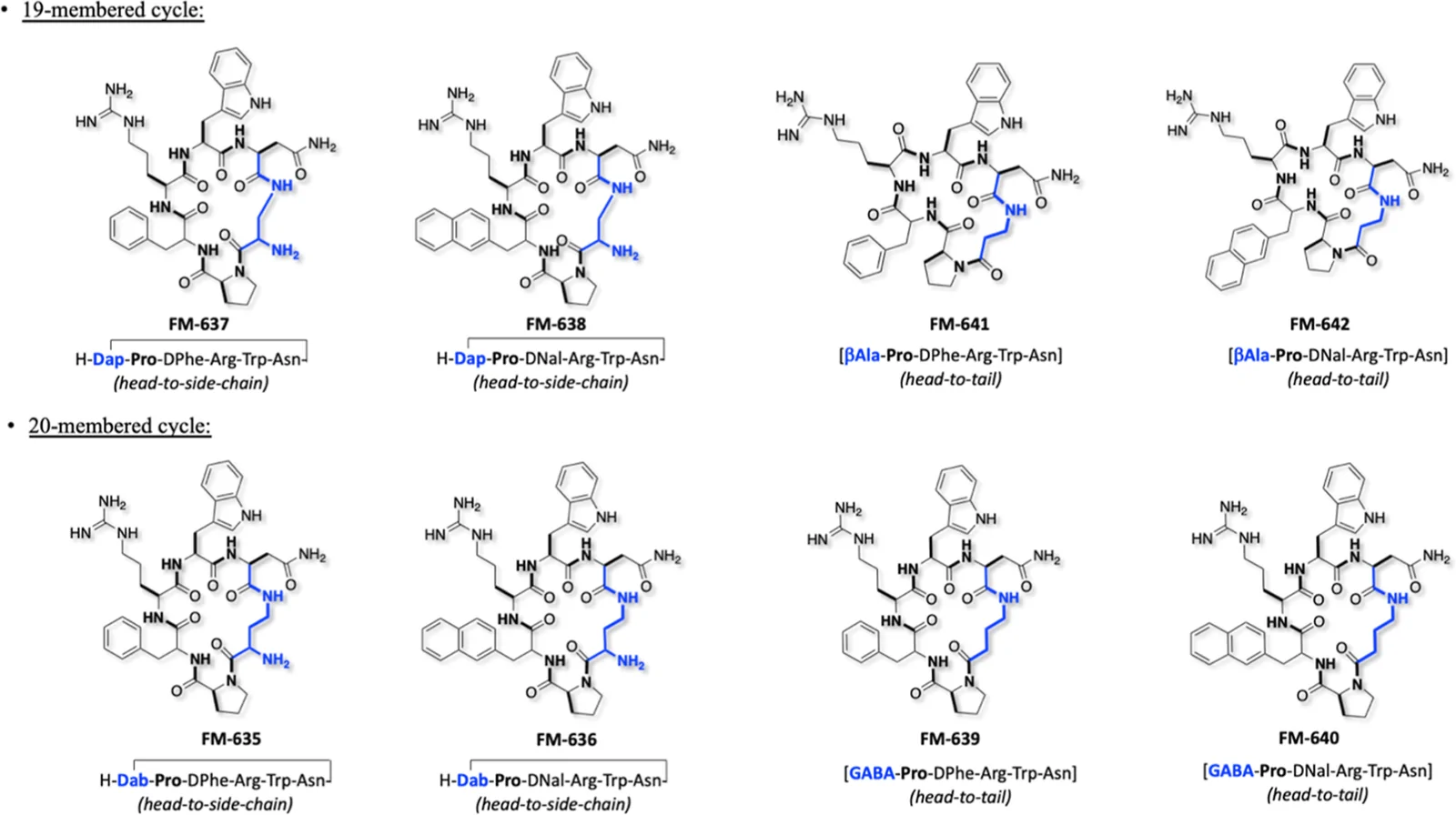
19-and 20-membered macrocyclic complexes.

**Figure 3:**
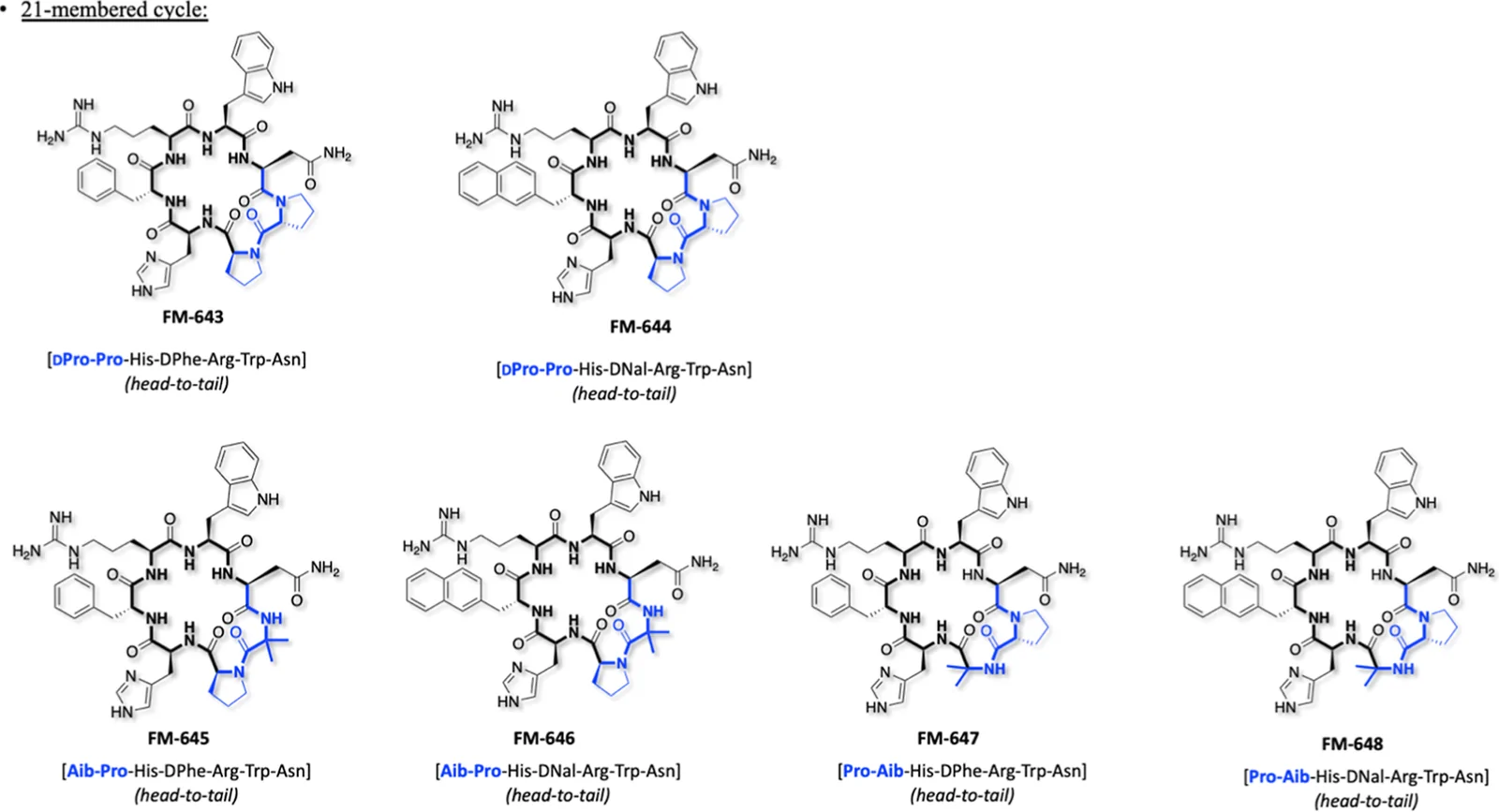
21-membered macrocycles.

The lactam bridge, which stabilizes the binding motif responsible for biological activity, has been engineered as either a head-to-side chain or head-to-tail, with additional conformational restrictions incorporated in some cases. These structural modifications were motivated by previous studies on analogous molecules, underscoring the importance of these features in determining the selectivity and potency toward melanocortin receptors.^31^ Although most compounds documented in the literature typically exhibit a 23-membered ring, previous studies have demonstrated that ligands with a 19-membered ring structure can maintain substantial activity,^32^ highlighting the potential of smaller ring structures.

Four peptide analogs related to the structures of **Pro** ^6^-**MT-II** and **PG-901** were characterized by the presence of a 20-membered ring (**FM635**, **FM636**, **FM639**, and **FM640**), in which the His residue was substituted with Pro, and the lactam bridge between Pro and Asn was created using Dab or GABA residues. The corresponding 19-membered ring structures (**FM637**, **FM638**, **FM641**, and **FM642**) containing Dap and β-Ala instead of Dab and GABA to form a smaller lactam bridge were also synthesized and evaluated. Six macrocyclic peptides analogous to **MT-II** and **SH-U9119** were prepared using 21-membered rings, including lactam bridges. Specifically, in peptides **FM643** and **FM644**, the lactam bridge primarily consisted of two proline residues (DPro-Pro), whereas in the other four compounds (**FM645**-**FM648**), the lactam bridge was formed by Aib-Pro or Pro-Aib combinations. The presence of these two amino acid residues can reduce the flexibility of the peptide chain, thereby affecting its overall conformation.

All compounds were tested on the four *h*MCRs to investigate their agonist activity, and inactive compounds were further screened for potential antagonistic activity. The results are shown in Table 1. Peptides **FM635** and **FM636** were selected for detailed conformational studies because of their remarkable pharmacological profiles as potent and selective MC4 receptor agonists and antagonists. To gain structural insights into these peptides, their conformations were analyzed using NMR spectroscopy in an aqueous solution.

**Table 1:** pEC_50_ and pIC_50_ Values of Macrocyclic Peptides on Melanocortin Receptors^a^

			*h*MC1R	*h*MC3R		*h*MC4R		*h*MC5R	
code	ring-size	peptide sequence	pEC_50_ ± SD	pEC_50_ ± SD	pIC_50_ ± SD	pEC_50_ ± SD	pIC_50_ ± SD	pEC_50_ ± SD	pIC_50_ ± SD
MT-II	23	Ac-Nle-[Asp-His-DPhe-Trp-Lys]-NH2	10.3 ± 0.40	10.1 ± 0.22		10.2 ± 0.25		9.1 ± 0.34	
SHU-9119	23	Ac-Nle-[Asp-His-D(2)Nal-Trp-Lys]-NH2	8.2 ± 0.06		8.47 ± 0.19		9.58 ± 0.11	8.5 ± 0.10	
PG-901	23	Ac-Nle-[Asp-Pro-D(2)Nal-Trp-Lys]-NH2	7.8 ± 0.30		8.1 (pA_2_)		9.6 (pA_2_)	10.1 ± 0.10	
FM635	20	H-[Dab-Pro-DPhe-Arg-Trp-Asn]	6.67 ± 0.02	0%^b^	0%^b^	8.1 ± 0.12	-	81%^b^	0%^b^
FM636	20	H-[Dab-Pro-DNal-Arg-Trp-Asn]	6.38 ± 0.07	0%	82%^b^	0%^b^	8.52 ± 0.00	8.0 ± 0.10	-
FM637	19	H-[Dap-Pro-DPhe-Arg-Trp-Asn]	6.5 ± 0.12	0%^b^	0%^b^	7.1 ± 0.25	-	63%^b^	0%^b^
FM638	19	H-[Dap-Pro-D(2)Nal-Arg-Trp-Asn]	7.2 ± 0.06	0%^b^	67%^b^	0%^b^	8.07 ± 0.03	7.4 ± 0.14	-
FM639	20	[GABA-Pro-DPhe-Arg-Trp-Asn]	7.08 ± 0.04	33%^b^	0%^b^	7.5 ± 0.07	-	84%^b^	0%^b^
FM640	20	[GABA-Pro-D(2)Nal-Arg-Trp-Asn]	6.5 ± 0.08	0%^b^	77%^b^	0%^b^	7.87 ± 0.10	7.4 ± 0.02	-
FM641	19	[βAla-Pro-DPhe-Arg-Trp-Asn]	6.1 ± 0.11	0%^b^	0%^b^	6.7 ± 0.27	-	0%^b^	0%^b^
FM642	19	[βAla-Pro-D(2)Nal-Arg-Trp-Asn]	7.2 ± 0.01	0%^b^	56%^b^	0%^b^	7.8 ± 0.07	7.5 ± 0.02	-
FM643	21	[DPro-Pro-His-DPhe-Arg-Trp-Asn]	8.1 ± 0.41	6.8 ± 0.47	0%^b^	8.6 ± 0.04	-	36%^b^	0%^b^
FM644	21	[DPro-Pro-His-D(2)Nal-Arg-Trp-Asn]	8.0 ± 0.08	0%^b^	6.27 ± 0.6	10%^b^	8.12 ± 0.03	7.6 ± 0.16	-
FM645	21	[Aib-Pro-His-DPhe-Arg-Trp-Asn]	7.5 ± 0.08	6.5 ± 0.01	0%^b^	8.2 ± 0.11	-	3%^b^	0%^b^
FM646	21	[Aib-Pro-His-D(2)Nal-Arg-Trp-Asn]	7.3 ± 0.02	0%^b^	50%^b^	0%^b^	7.13 ± 0.22	7.00 ± 0.00	-
FM647	21	[Pro-Aib-His-DPhe-Arg-Trp-Asn]	7.7 ± 0.13	6.5 ± 0.14	0%^b^	8.4 ± 0.15	-	22%^b^	0%^b^
FM648	21	[Pro-Aib-His-D(2)Nal-Arg-Trp-Asn]	7.7 ± 0.11	0%^b^	6.31 ± 0.4	0%^b^	8.38 ± 0.04	8.1 ± 0.25	-

a Compounds were screened from 1 μM-10 pM in 10-fold serial dilutions. The IC_50_ is the antagonism of MT-II at EC_80–90_ concentrations. (*N* = 2).

b % values indicate activity at 1 μM. Figure S18 shows a bar chart illustrating the selectivity between agonist and antagonist activities of the synthesized compounds.

### Structure–Activity Relationship Analysis

The potency of the synthesized compound library was reduced relative to that of the reported parent compounds, **MT-II**, **SHU-9119**, and **PG-901** (Table 1). This outcome was anticipated, given the structural modifications, particularly the absence of Ac-Nle, a common component of MT-II-like compounds that contributes to receptor binding. The selectivity profiles of the other MT-II analogs, specifically for MC1R, MC3R, MC4R, and MC5R, were generally consistent with expectations but exhibited some notable characteristics. All compounds displayed the strongest activity at MC4R, functioning as either agonists (containing DPhe) or antagonists (containing D(2′)­Nal). Compounds exhibiting agonist activity against MC1R had pEC_50_ values ranging from 8.1 to 6.1. Compounds with D(2′)­Nal residues demonstrated agonist activity at MC5R, whereas those with DPhe residues exhibited weak activity on the same receptor. Regarding MC3R activity, compounds with DPhe showed weak or no agonist activity, whereas those with a D(2′)­Nal residue exhibited modest antagonistic activity. Certain trends have been observed in the receptor series for the development of specific SAR subtypes.

For *h*MC1R, His^6^-containing **FM643** and **FM644** displayed the highest pEC_50_ values (8.1 and 8.0, respectively), indicating a potency comparable to that of the peptides SHU-9119 and PG-901. Additionally, these two peptides acted as agonist and antagonist at *h*MC4R, respectively (pEC_50_ 8.6 and pIC_50_ 8.1). **FM645**-**FM648**, containing Pro-Aib/Aib-Pro, exhibited reduced agonist activity at *h*MC1R (approximately pEC_50_ = 7.5), likely because of the different conformations of the lactam bridge. The remaining eight compounds examined in this study, which did not contain a His^6^ residue, exhibited relatively low activity (pEC_50_ = 6.5) in the assays performed. This behavior suggests that the presence of the His^6^ residue combined with a 21-membered ring size is favorable for enhanced agonist activity toward MC1R.

For *h*MC3R, most compounds exhibited no agonistic activity. However, **FM639** showed marginal effects, with 36% activity at 1 μM, whereas **FM643**, **FM645**, and **FM647** demonstrated low agonist activity (approximately pEC_50_ = 6.5). The antagonistic activities of the 14 compounds against *h*MC3R were also evaluated. Compounds containing D(2′)­Nal displayed moderate antagonist activity (pIC_50_ > 50% at 1 μM), consistent with previous research indicating that D(2′) Nal induces antagonistic effects (17). Among them, **FM644** and **FM648** exhibited the highest antagonistic activity (pIC_50_ 6.3).

For *h*MC4R, an increase in ring size leads to greater potency as an agonist. **FM635** exhibited notable selectivity, being approximately 20-fold less potent for MC1R (pEC_50_ 8.1 vs 6.67) and ∼50-fold less potent for MC5R (pEC_50_ 8.1 vs ∼ 6). D(2′)­Nal-containing compounds exhibited potent antagonistic activity against MC4R, consistent with prior findings that D(2′)­Nal substitutions convert agonists to antagonists (23). Most D(2′)­Nal-containing compounds had an approximate pIC_50_ value of 8.0 for *h*MC4R. Notably, the ring composition did not influence the antagonist potency, although **FM646** was approximately 10-fold weaker than **FM644**.

In the context of *h*MC5R, compounds incorporating DPhe demonstrated limited agonist activity, whereas most D(2′)­Nal-containing compounds exhibited moderate agonist activity, with a pEC_50_ value of approximately 7.5. Among these, **FM648** emerged as the most potent agonist, with a pEC_50_ value of 8.1, followed by **FM636**, with a pEC_50_ value of 8.0. Notably, no antagonistic activity was observed at *h*MC3R for peptides containing the D(2′)­Nal residue at the original positions of **SHU-9119** and **PG-901**. It is plausible that the ring dimensions, influenced by the conformational constraints imposed by the lactam bridge, are not conducive to interactions between the DPhe/D(2′)­Nal residues within the receptor pocket previously identified for *h*MC3R and *h*MC4R.

Further insights into the structure–activity relationship (SAR) of the synthesized compounds were obtained through pairwise comparisons. Table 2 presents a comparison of compounds with and without free amino groups on the lactam bridge. A detailed pairwise comparison of analogs **FM635**-**FM642** revealed that the presence of a positively charged amino group within the bridge was consistently associated with enhanced potency at MC4R, while producing mixed effects at MC1R, MC3R, and MC5R.

**Table 2:** Pairwise Comparisons of Macrocycles with and Without Amino Groups on the Lactam Bridge^a^

		*h*MC1R	*h*MC3R		*h*MC4R		*h*MC5R	
name	peptide sequence	pEC_50_ ± SD	pEC_50_ ± SD	pIC_50_ ± SD	pEC_50_ ± SD	pIC_50_ ± SD	pEC_50_ ± SD	pIC_50_ ± SD
FM635	H-[Dab-Pro-DPhe-Arg-Trp-Asn]	6.67 ± 0.02	0%^b^	0%^b^	8.1 ± 0.12	-	81%^b^	0%^b^
FM639	[GABA-Pro-DPhe-Arg-Trp-Asn]	7.08 ± 0.04	33%^b^	0%^b^	7.5 ± 0.07	-	84%^b^	0%^b^
FM636	H-[Dab-Pro-DNal-Arg-Trp-Asn]	6.38 ± 0.07	0%^b^	82%^b^	0%^b^	8.52 ± 0.00	8.0 ± 0.10	-
FM640	[GABA-Pro-DNal-Arg-Trp-Asn]	6.5 ± 0.08	0%^b^	77%^b^	0%^b^	7.87 ± 0.10	7.4 ± 0.02	-
FM637	H-[Dap-Pro-DPhe-Arg-Trp-Asn]	6.5 ± 0.12	0%^b^	0%^b^	7.1 ± 0.25	-	63%^b^	0%^b^
FM641	[βAla-Pro-DPhe-Arg-Trp-Asn]	6.1 ± 0.11	0%^b^	0%^b^	6.7 ± 0.27	-	0%^b^	0%^b^
FM638	H-[Dap-Pro-DNal-Arg-Trp-Asn]	7.2 ± 0.06	0%^b^	67%^b^	0%^b^	8.07 ± 0.03	7.4 ± 0.14	-
FM642	[βAla-Pro-DNal-Arg-Trp-Asn]	7.2 ± 0.01	0%^b^	56%^b^	0%^b^	7.8 ± 0.07	7.5 ± 0.02	-

a Compounds were screened from 1 μM-10 pM in 10-fold serial dilutions. The IC_50_ is the antagonism of MT-II at the EC_80–90_ concentration. (*N* = 2).

b % values indicate activity at 1 μM.

For the 19-membered compounds (**FM637** vs **FM641** and **FM638** vs **FM642**), those containing DPhe exhibited reduced activity on the *h*MC1R compared to the reference compounds. Conversely, compounds with the same ring sizes at 19 members but incorporating D(2′)­Nal demonstrated increased *h*MC1R activity. Similarly, the presence of an amino group on the side of the lactam bridge in D(2′)­Nal-containing compounds enhanced their antagonistic potency at *h*MC4R, with a more pronounced effect observed in **FM636**, which was characterized by a ring size of 20. A comparable pattern was observed for the compound pairs **FM636**-**FM638** and **FM640**-**FM642** on *h*MC5R. Notably, for these series of compounds, the amino group on the lactam bridge exhibited a greater influence on the affinity or potency for the *h*MC4 receptor than for the *h*MC1R.

The data set derived from the synthesized compounds corroborated the typical activity profiles of macrocyclic peptides containing DPhe or D(2′)­Nal at position 7. The significance of these residues in determining agonist or antagonist activity is consistent with the results obtained for compounds with similar ring sizes. Furthermore, these findings suggest that the presence of a Pro^6^ residue may enhance receptor selectivity compared to other MT-II analogs, indicating that targeted modifications at this position, combined with conformational constraints in the lactam bridge, could lead to improved selectivity in other analogs.

Table 3 presents a comparison of compounds with 21-membered macrocyclic peptides on *h*MCRs. A detailed pairwise comparison of analogs **FM643**-**FM648** revealed the typical activity profiles of macrocyclic peptides containing DPhe or D(2′)­Nal at position 7, although less potent but more selective at *h*MC4R.

**Table 3:** pEC_50_ and pIC_50_ Values of 21-Membered Macrocyclic Peptides on *h*MCRs^a^

		*h*MC1R	*h*MC3R		*h*MC4R		*h*MC5R	
Name	peptide sequence	pEC_50_ ± SD	pEC_50_ ± SD	pIC_50_ ± SD	pEC_50_ ± SD	pIC_50_ ± SD	pEC_50_ ± SD	pIC_50_ ± SD
FM643	[DPro-Pro-His-DPhe-Arg-Trp-Asn]	8.1 ± 0.41	6.8 ± 0.47	0%^b^	8.6 ± 0.04	-	36%^b^	0%^b^
FM645	[Aib-Pro-His-DPhe-Arg-Trp-Asn]	7.5 ± 0.08	6.5 ± 0.01	0%^b^	8.2 ± 0.11	-	3%^b^	0%^b^
FM647	[Pro-Aib-His-DPhe-Arg-Trp-Asn]	7.7 ± 0.13	6.5 ± 0.14	0%^b^	8.4 ± 0.15	-	22%^b^	0%^b^
FM644	[DPro-Pro-His-DNal-Arg-Trp-Asn]	8.0 ± 0.08	0%^b^	6.27 ± 0.6	10%^b^	8.1 ± 0.03	7.6 ± 0.16	-
FM646	[Aib-Pro-His-DNal-Arg-Trp-Asn]	7.3 ± 0.02	0%^b^	50%^b^	0%^b^	7.1 ± 0.22	7.0 ± 0.00	-
FM648	[Pro-Aib-His-DNal-Arg-Trp-Asn]	7.7 ± 0.11	0%^b^	6.31 ± 0.4	0%^b^	8.4 ± 0.04	8.1 ± 0.25	-

a Compounds were screened from 1 μM-10 pM in 10-fold serial dilutions. The IC_50_ represents the antagonism of MT-II at the EC_80_-_90_ concentration. (*N* = 2).

b % values indicate activity at 1 μM.

### Competitive Antagonism Analysis of FM636 and FM648 at MC4R

The above results relating to antagonistic behavior measured by pIC_50_ do include an assumption that the antagonism of MC4R is competitive. To confirm this, **FM636** and **FM648** were further assessed using the cAMP antagonist assay (Schild analysis and pA_2_ measurement). **FM636** yielded pA_2_ = 8.29 with a Schild slope of 1.0, while **FM648** showed pA_2_ = 7.81 (Table 4). In comparison to **SHU-9119**, both **FM636** and **FM648** demonstrated slightly lower potency but were selective for hMC4R. Despite these slightly lower values compared to **SHU-9119**, both compounds are still within the range of high-affinity competitive antagonists, indicating that they interact with the MC4R binding site in a reversible and competitive manner.

**Table 4:** pA_2_ of FM636 and FM 648 Compounds on hMC4R^a^

	FM636	FM648	SHU9119
pA_2_	8.29	7.81	9.44
Schild slope	1.00	1.17	1.24

a Compounds were screened from 1 μM- 100 fM in 10-fold dilutions.

### Binding Affinity Analysis of Selected MC4R and MC1R Ligands

Finally, a fluorescence-activated cell sorting (FACS)-based competitive binding assay was used to evaluate the receptor-binding affinities of the selected compounds.^33^ While functional assays, such as cAMP measurements, quantify efficacy and potency, reflecting a compound’s ability to activate or inhibit receptor signaling, binding assays directly measure affinity, which describes how strongly a ligand interacts with the receptor, independent of downstream signaling. **FM635**, **FM636**, **FM643**, and **FM644** were assessed for their affinities toward MC4R and MC1R (Table 5). These compounds were selected based on their previously demonstrated potent agonist (pEC_50_) or antagonist (pIC_50_) activities in functional assays.

**Table 5:** pEC_50_ and pKi of FM’s Compounds on *h*MC1R and *h*MC4R^a^

	*h*MC1R	*h*MC1R	*h*MC4R	*h*MC4R	*h*MC4R
	agonist	binding	agonist	antagonist	binding
name	pEC_50_ ± SD	pKi ± SD	pEC_50_ ± SD	pIC_50_ ± SD	pKi ± SD
MT-II	11.5 ± 0.35	10.1 ± 0.29	10.9 ± 0.09	-	9.3 ± 0.27
FM635	6.7 ± 0.02	65%^b^	8.1 ± 0.12	-	7.5 ± 0.02
FM636	6.4 ± 0.07	7.0 ± 0.14	0%^b^	8.5 ± 0.00	9.4 ± 0.09
FM643	8.1 ± 0.41	8.1 ± 0.10	8.6 ± 0.04	-	7.8 ± 0.06
FM644	8.0 ± 0.08	8.4 ± 0.06	10%^b^	8.1 ± 0.03	8.8 ± 0.03

a Compounds were screened from 1 μM–100 fM in 10-fold dilutions. (*N* = 2).

b % values indicate activity at 1 μM.

Among these analogues, at MC4R, **FM636** exhibited the strongest binding (p*K_i_* = 9.4), followed by **FM644** (p*K_i_* = 8.8), **FM643** (p*K_i_* = 7.8), and **FM635** (p*K_i_* = 7.5). At MC1R, **FM643** and **FM644** displayed the highest binding affinities (p*K_i_* ≈ 8.0), whereas **FM635** and **FM636** showed lower affinities (pK_*i*_ = 65% and 7, respectively). Comparison across receptors revealed that **FM635**, **FM636**, and **FM644** bind more strongly to MC4R than to MC1R, whereas **FM643** displayed comparable affinity at both receptors. Overall, the binding affinities varied by approximately two log units across the series, demonstrating substantial variation in receptor engagement driven by structural modifications.

The compounds showed good alignment between binding affinity and potency. For example, **FM643** and **FM644** exhibited both high MC1R binding affinity and strong agonist potency (pEC_50_ ≈ 8.0), whereas **FM636** and **FM644** displayed strong MC4R binding affinity and potent antagonist activity (pIC_50_ = 8.5 and 8.1). However, it was notable that at MC4R, the binding affinity (pKi) was lower than the agonist efficacy (pEC_50_), whereas for the antagonists, the binding affinity (pKi) was higher than the antagonist pIC_50_.

These results established a clear correlation between functional potency and receptor-binding affinity; compounds exhibiting stronger biological activity also possessed higher receptor-binding strength. Notably, the MC4R antagonists in this series generally exhibited higher binding affinities than the agonists, even when their overall potencies were comparable. This pattern suggests subtle differences in the receptor–ligand interaction dynamics between agonist- and antagonist-bound receptor conformations.

### Conformational Analysis of Selected Peptides

Peptides **FM635** and **FM636** were selected for detailed conformational studies because of their remarkable pharmacological profiles as a potent and selective *h*MC4 receptor agonist and antagonist, respectively. To gain structural insights into these peptides, their conformations were analyzed using NMR spectroscopy in an aqueous solution. The NMR spectra of **FM635** and **FM636** were significantly similar, characterized by multiple duplicate signals indicative of conformational heterogeneity. Comprehensive ^1^H NMR chemical shift assignments for the predominant conformer of each peptide were successfully achieved (Tables S2 and S3, Supporting Information). The absence of diagnostic NOE cross-peaks in the NOESY spectra suggests a degree of conformational flexibility, despite the cyclic nature of both peptides. Upfield shifts in the Arg^8^ proton signals were observed, consistent with the shielding effects of the aromatic rings of DPhe/DNal^7^ and Trp,^9^ as previously reported for other melanocortin peptides.^26^

The structure of **FM635** and **FM63**6 was calculated using simulated annealing under NMR-derived conformational restraints (Tables S4 and S5, Supporting Information). A multistate ensemble approach was employed to account for the peptide flexibility.^34^ The resulting structures were clustered according to backbone similarity, and the ten lowest-energy conformers from the most populated cluster for each peptide are shown in Figure 4A,B.

**Figure 4:**
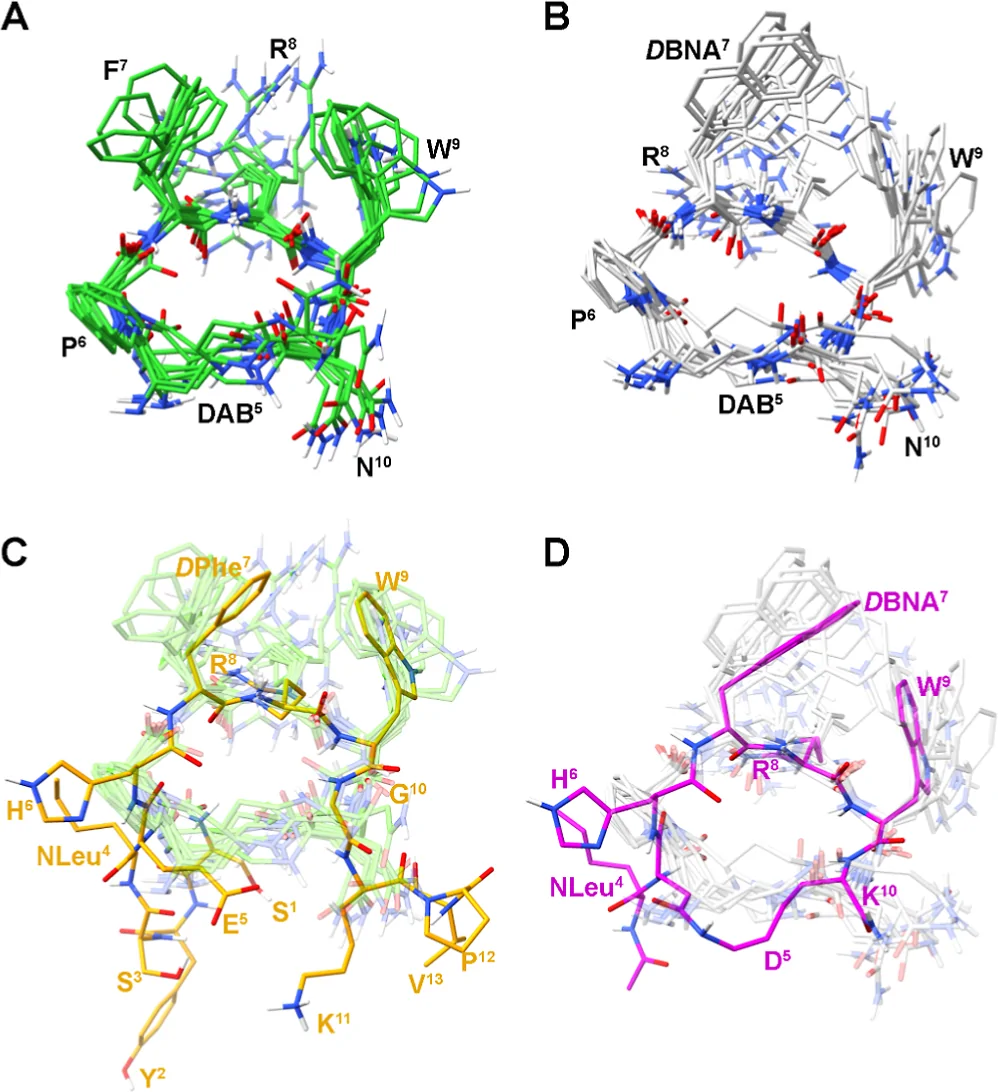
(A) Superposition of an ensemble of ten NMR-derived structures of **FM635**. Atoms are colored by atom type (carbon, green; nitrogen, blue; oxygen, red; hydrogen, white). (B) Superposition of an ensemble of ten NMR-derived structures of **FM636** (color code as in panel A, except carbon atoms, which are shown in gray). (C) Superposition of the **FM635** ensemble (shown as shadows) and the NDP structure from its complex with MC4R (PDB code: 7PIV) (color code as in panel A, except carbon atoms, which are shown in yellow). (D) Superposition of the **FM636** ensemble (shown as shadows) and **SHU-9119** from its complex with MC4R (PDB code: 6W25) (color code as in panel A, except carbon atoms, which are shown in violet). For clarity, only polar hydrogens are shown.

In Figure 4C, the **FM635** conformers are superimposed on the known agonist NDP, whereas the **FM636** conformers are overlaid with antagonist **SHU-9119** (Figure 4D). The reference ligand structures were obtained from the crystal complexes of these peptides with MC4R. Both **FM635** and **FM636** exhibited structural resemblance to their respective reference compounds within the pharmacophore region, with backbone RMSD values below 1.0 Å (residues 6–9, peptide position number based on α-MSH) and heavy-atom RMSD values below 2.0 Å (residues 7–9).

Notably, the structural conformation of known peptides, whether agonists or antagonists, bound to various melanocortin receptors (MC1R, MC3R, MC4R, and MC5R) exhibited significant similarity in the pharmacophore region (residues 6–9), as illustrated in Figures S16 and S17 of the Supporting Information. This observation suggests that the peptides under investigation are likely to adopt similar conformations when they are bound to MCRs. In this context, the high similarity of the most populated conformational clusters with the receptor-bound crystal structures of known agonists for **FM635** and antagonists for **FM636** indicated that both peptides assume conformations in solution that are compatible with receptor binding. To further test this hypothesis, the lowest-energy NMR conformer of **FM635** was docked into *h*MC3R and *h*MC4R receptor models. The best-scored poses are shown in Figure 5. In both complexes, **FM635** was predicted to occupy the orthosteric binding pocket located among TM3–TM7 and the extracellular loops EL1–EL3 (Figure 5A,B). The main predicted ligand–receptor interactions for the **FM635**–*h*MC3R and **FM635**–*h*MC4R complexes are shown in Figure 5C,D, respectively. The docking energies reported in Table S6 of the Supporting Information suggested a more favorable interaction of **FM635** with *h*MC4R than with *h*MC3R, with total binding energies of −136.8 and −106.1 kcal/mol, respectively, in agreement with the pharmacological data.

**Figure 5:**
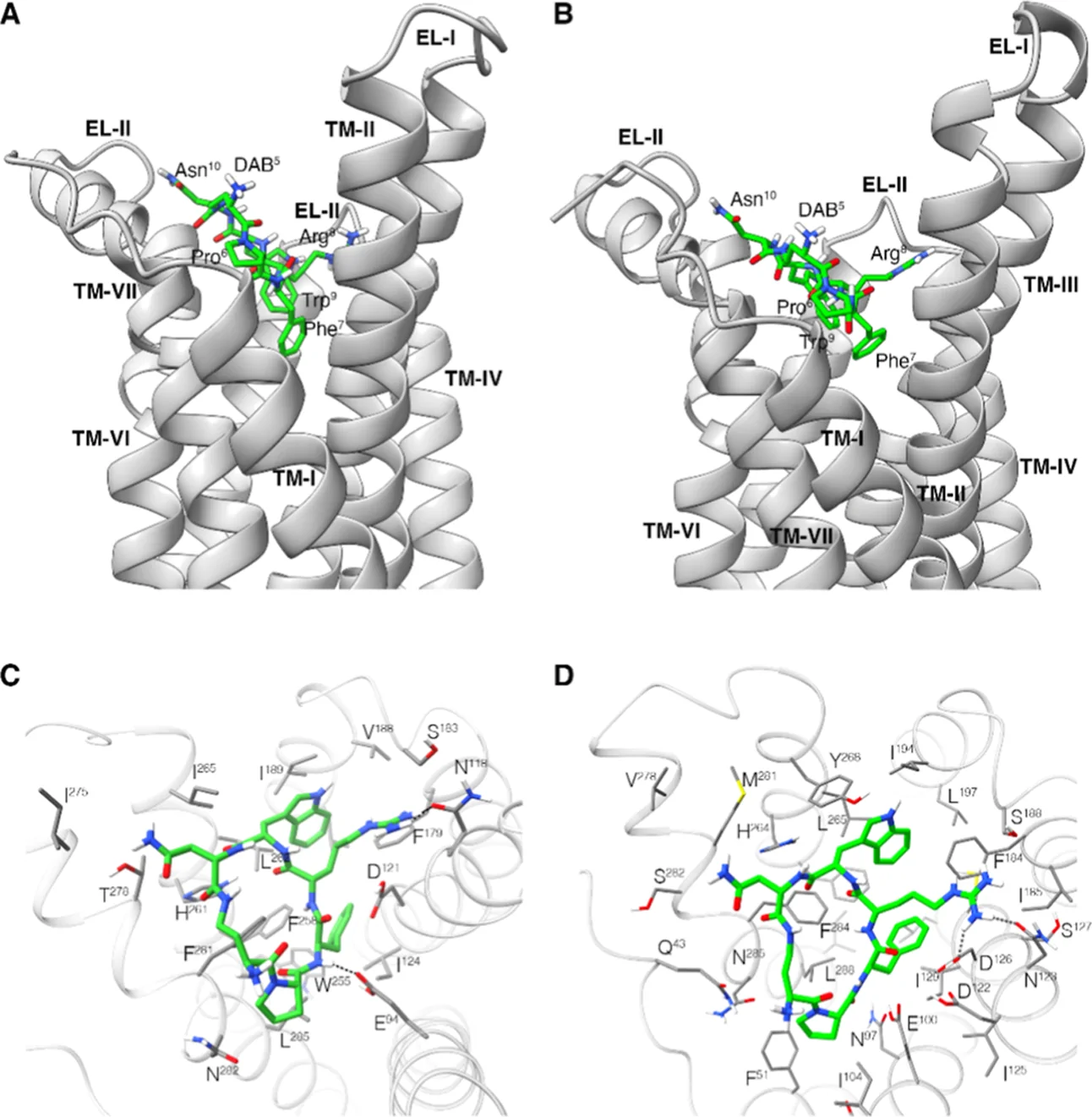
*h*MC3R (A) or *h*MC4R (B) model complexed with **FM635**. Heavy atoms of **FM635** (carbon, green; nitrogen, blue; oxygen, red; polar hydrogens, white). Receptor backbone is represented in gray ribbon. **FM635** within the binding pocket of *h*MC3R (C) or *h*MC4R (D). Hydrogen bonds are represented with dashed lines.

Beyond this observation, the imperfect overlap between the solution structures of **FM635**/**FM636** and the receptor-bound conformations of the reference ligands (Figure 4) suggests the occurrence of a subsequent induced-fit mechanism upon receptor binding. Variations in the ability of the receptors to facilitate such conformational adaptation may further account for the observed selectivity of **FM635** and **FM636** for MC4R compared to MC3R. Alternatively, the structural differences in the *N*-terminal regions of MC3R and MC4R, which are not conserved, may contribute to this selective behavior.

Notably, a disulfide bond between Cys^35^ in the *N*-terminus and Cys^276^ in the third extracellular loop was present only in MC3R, potentially imparting greater rigidity to the *N*-terminus. Combined with the intrinsic rigidity of 20-membered cyclic peptides, particularly when the pharmacophore region is constrained by receptor tethering, this rigidity may impede compensatory adjustments upon binding, resulting in reduced compatibility with MC3R. As a matter of fact, all novel analogs featuring a reduced ring size (19–21 atoms) relative to the classical melanocortin peptide ligands **MT-II**, **SHU-9119**, and **PG-901** (23 atoms), exhibited diminished activity at MC3R. However, further experimental and computational studies are necessary to elucidate the molecular determinants of selectivity.

Overall, the macrocyclic peptides exhibited varying degrees of potency and selectivity, with certain analogs displaying a marked preference for MC4R. Compared with existing melanocortin receptor agonists and antagonists, our compounds demonstrated enhanced selectivity, potentially leading to fewer off-target effects in therapeutic applications. These findings suggest a promising direction for the design of next-generation melanocortin receptor-targeted therapies that exploit the structural advantages of these macrocyclic peptides. Taken together, these data place the present midsized macrocycles in a distinct position relative to the classical melanocortin templates **MT-II**, **SHU-9119**, and **PG-901**. Although the reduction of ring size from the classical 23-membered macrocycle to 19–21-membered systems generally decreased overall potency, it also provided a useful handle to modulate receptor subtype selectivity. In particular, the Pro6-containing 20-membered analogues **FM635** and **FM636** retained strong MC4R activity while showing markedly reduced activity at MC3R and limited activity at MC5R. The DPhe/D(2′)­Nal switch preserved the expected agonist-to-antagonist functional transition at MC4R, while the nature of the lactam bridge and the presence of a positively charged amino group further influenced potency and selectivity. These findings indicate that controlled contraction of the macrocyclic framework can be exploited as a design strategy to bias melanocortin ligands toward MC4R-selective pharmacological profiles.

## Conclusion

This study identified a new series of midsized macrocyclic peptides with distinctive selectivity profiles toward human melanocortin receptors. Although contraction of the classical 23-membered melanocortin macrocycle to 19–21-membered systems generally reduced overall potency compared with the parent ligands **MT-II**, **SHU-9119**, and **PG-901**, it also provided a valuable strategy to modulate receptor subtype selectivity. In particular, the data highlight three main structural determinants: the DPhe/D(2′)­Nal switch as a key driver of agonist versus antagonist behavior at *h*MC4R, the presence of Pro^6^ as a favorable element for receptor subtype discrimination, and the nature of the lactam bridge, including ring size and the presence of a positively charged amino group, as an important modulator of MC4R potency and selectivity.

Among the synthesized analogues, **FM636** and **FM648** emerged as potent *h*MC4R antagonists, whereas **FM635** displayed selective *h*MC4R agonist activity. Additionally, **FM636** and **FM648** were identified as selective ligands for *h*MC5R. Notably, the 20-membered Pro^6^-containing analogues **FM635** and **FM636** showed reduced activity at *h*MC3R and limited off-target activity at *h*MC5R, supporting the concept that controlled contraction of the macrocyclic framework can bias melanocortin ligands toward MC4R-selective pharmacological profiles. Overall, this “Goldilocks-inspired” approach provides useful SAR insights for the optimization of midsized macrocycles as selective melanocortin receptor ligands and potential leads for therapeutic applications in metabolic and inflammatory diseases.

## Experimental Section

### Materials and General Procedures

*N* ^*a*^-Fmoc-protected amino acids such as Fmoc-Arg­(Pbf)–OH, Fmoc-Asp-*O*All, Fmoc-His­(Trt)–OH, Fmoc-Pro-OH, and Fmoc-Trp­(Boc)–OH were purchased from IRIS Biotech GmbH (Marktredwitz, Germany). Unconventional amino acids, such as Fmoc-Aib–OH, Fmoc-Dab­(Alloc)–OH, Fmoc-Dap­(Alloc)–OH, Fmoc-GABA–OH, Fmoc-β-Ala-OH, Fmoc-DPhe-OH, Fmoc-DPro–OH, and Fmoc-DNal(2′)–OH, were obtained from IRIS Biotech GmbH (Marktredwitz, Germany), GL Biochem Ltd. (Shanghai, China)­and Fluorochem (Hadfield, United Kingdom). Coupling and additive reagents, including morpholino-carbenium hexafluorophosphate (COMU), ethyl cyano­(hydroxyimino)­acetate (Oxyma), and 7-azabenzotriazol-1-yloxy) trispyrrolidinophosphonium hexafluorophosphate (PyAOP), were purchased from Merck Life Science (Milan, Italy). Fmoc-Rink amide resin (loading 0.63 mmol/g), *N,N*-diisopropylethylamine (DIPEA), piperidine, trifluoroacetic acid (TFA) and triisopropylsilane (TIS) were obtained from Merck Life Science (Milan, Italy). Additional reagents, including 1,3-dimethylbarbituric acid (NDMBA), tetrakis­(triphenylphosphine)­palladium(0) [Pd­(PPh_3_)_4_], and sodium *N*,*N*-diethyldithiocarbamate, were also purchased from Merck Life Science (Milan, Italy). The solvents used for peptide synthesis, such as *N,N*-dimethylformamide (DMF), dichloromethane (DCM), and diethyl ether (Et_2_O), as well as solvents for HPLC analyses and purification (water, MeOH, and MeCN), were of reagent grade, obtained from Merck Life Science or VWR (Milan, Italy), and used without further purification. Anhydrous DMF and DCM were purchased from Merck Life Science (Milan, Italy).

Peptide purification was performed using RP-HPLC with a Shimadzu LC-8A preparative system equipped with a Phenomenex Kinetex C18 column (5 μm, 100 Å, 150 × 21.2 mm). Linear gradients of MeCN (0.1% TFA) in water (0.1% TFA), from 10 to 90% over 30 min, were applied at a flow rate of 10 mL/min with UV detection at 220 nm. The collected fractions were pooled, concentrated by rotary evaporation to remove MeCN, and lyophilized to obtain the final product. Analytical UHPLC was performed on a Shimadzu Nexera LC-30AD system using a Phenomenex Kinetex C18 column (5 μm, 100 Å, 150 × 4.6 mm) at a flow rate of 1 mL/min, with a gradient of MeCN (0.1% TFA) in water (0.1% TFA) from 10 to 90% over 15 min, with UV detection at 220 nm. All compounds evaluated in the biological assays exhibited purities >97% (Figures S1–S14), and their identities were confirmed by ESI-MS (Table S1 and Figure S15).

### Peptide Synthesis

Macrocyclic peptides were synthesized in a stepwise manner using solid-phase peptide synthesis (SPPS). Linear precursor sequences were assembled using Fmoc-based ultrasound-assisted SPPS (US-SPPS).^35^ Each peptide was synthesized on Fmoc-Rink amide resin (0.2 mmol, loading 0.63 mmol/g, 100–200 mesh). The resin was placed in a plastic syringe reactor and swollen in DMF for 20 min on an automated shaker. Fmoc deprotection was performed using 20% piperidine in DMF (0.5 + 1 min), with the syringe reactor placed in an ultrasonic bath. The resin was washed with DMF (2 mL × 3) and DCM (2 mL × 3). Coupling reactions were carried out using Fmoc-amino acid (2 equiv), COMU (2 equiv), Oxyma (2 equiv), and DIPEA (4 equiv) in DMF, and the mixture was subjected to ultrasonic irradiation for 10 min. After each deprotection and coupling step, the resin was washed with DMF (2 mL × 3) and DCM (2 mL × 3) sequentially. The reaction progress was monitored using the Kaiser or Chloranil tests to detect resin-bound primary and secondary amines, respectively.

#### Lactam Cyclization

Following the elongation of the linear precursor, allyl-based protecting groups were selectively removed according to previously established protocols.^36,37^ In brief, the resin was washed with dichloromethane (DCM) (3 × 2 mL) and subsequently dried under a vacuum. The resin was then treated with a suspension of Pd­(PPh_3_)­4 (0.15 equiv) and *N,N′*-dimethylbarbituric acid (NDMBA) (3 equiv) in a dry DCM/DMF mixture (3:2, v/v) and agitated for 1 h. The resin was filtered and washed with dimethylformamide (DMF) (2 mL × 3) and DCM (2 mL × 3), and the deprotection step was repeated. Subsequently, the resin was treated with a 0.5% solution of sodium *N,N′*-diethyldithiocarbamate in DMF and agitated for 30 min, then washed with DMF (2 mL × 3) and DCM (2 mL × 3). The completion of allyl deprotection was confirmed by liquid chromatography–mass spectrometry (LC–MS) analysis of a cleaved resin aliquot (5 mg). At this stage, the macrocyclization strategy was contingent on the type of cyclization. For head-to-side cyclization, peptides **FM635-FM638** possessed a released amine and carboxylic acid following allyl deprotection. Intramolecular cyclization was conducted on the resin using PyAOP (2 equiv) and DIPEA (4 equiv) in DMF for 16 h on an automated shaker. Successful cyclization was confirmed by LC–MS analysis of a cleaved resin aliquot [5 mg treated with 1 mL of trifluoroacetic acid/triisopropylsilane/water (TFA/TIS/H_2_O) (95:2.5:2.5, v/v/v)]. Finally, the *N*-terminal Fmoc group was removed, and the resin was washed with DCM (2 mL × 3) and dried under vacuum. For head-to-tail cyclization, peptides **FM639**-**FM648** presented a free carboxylic acid after allyl deprotection. The resin was treated with 20% piperidine in DMF to remove the *N*-terminal Fmoc group, and intramolecular cyclization was subsequently performed on the resin using PyAOP (2 equiv) and DIPEA (4 equiv) in DMF for 16 h on an automated shaker. Successful cyclization was confirmed by LC–MS analysis of the cleaved resin aliquot (5 mg). The resin was washed with DCM (2 mL × 3) and dried under vacuum. Finally, for all peptides, the resin was cleaved, and the protecting groups were removed by treatment with TFA/TIS/H_2_O (95:2.5:2.5, v/v/v) for 3 h. Following resin filtration, the crude peptide was precipitated with cold diethyl ether (Et_2_O), collected, and dried under vacuum.

### Pharmacology

Assays of MCR function and binding affinity were conducted as previously described using Human Embryonic kidney 293 (HEK293) cells were used that stably express isoforms of MCR together with a Hygromycin B resistance gene. In brief, cells were seeded into 96-well, flat bottom, clear plates (15,000/well) with growth media (100 μL), then incubated for 24 h at 37 °C and 5% CO_2_. The test peptides were dissolved in DMSO beforehand. After 24 h, stimulation buffer was prepared using Phenol red free DMEM (Gibco, Thermo Fisher Scientific, MA, USA), 0.1% m/v Bovine Serum Albumin (BSA) (Sigma-Aldrich, MO, USA) and the Phosphodiesterase Inhibitor 3-Isobutyl-1-Methylxanthine (IBMX) (Sigma-Aldrich, MO, USA) and prepared 1:10 dilution of compounds in stimulation buffer. Then, the cells were stimulated with the buffer for 30 min (90 μL) and test compound was added (10 μL), followed by a 45 min incubation. Afterward, using ice-cold absolute ethanol (Univar Solutions, Australia). to stop the reaction and allow to sit at room temperature overnight until all the ethanol has evaporated. Once all the ethanol has evaporated, the cells were lysed by treatment with lysis buffer (225 μL, pH 7.4) containing BSA (0.1%, w/v), Tween 20 (0.3%, v/v) (Sigma-Aldrich, MO, USA), and Hepes (5 mM).

The cAMP levels in the samples were quantified using the LANCE cAMP assay kit (Revvity, WM, US). Briefly, cell lysate (5 μL) with 10 μL of Eu-cAMP tracer solution (1:200 dilution of Eu-cAMP tracer stock in detector buffer) and 5 μL ULight anti-cAMP solution (1:294 dilution of ULight anti-cAMP stock in detector buffer) were transferred into a 384 well Optiplate (Revvity, WM, US) in reduced light conditions and incubated for 1 h at room temperature. cAMP signals were detected using an EnVision/PerkinElmer plate reader with excitation at 320 nm and emission at 615 nm.

To determine the properties of an antagonist of MT-II induced MCR activation, the assay was conducted as described above, and cells were coincubated with varying concentrations of the test compound (10 μL) and a fixed concentration of MT-II (10 μL). The fixed concentration of MT-II was determined based on the EC80–90 value for the corresponding receptor.

For competitive antagonism (Schild analysis), the assays were conducted as described above, but using a fixed concentration of the test compound in the presence of varying concentrations of MT-II (10 μL). The test compounds were assayed at 1000, 100, and 10 nM.

### Competition Binding Assays

To evaluate competition binding at MCRs, a Fluorescence-Activated Cell Sorting (FACS)-based assay was employed.^33^ Flp-In HEK293 cells stably expressing melanocortin receptors (MCRs) were cultured in T150 flasks with growth medium at 37 °C and 5% CO_2_. Upon reaching 80% confluency, the cells were detached by using the Tryple Express enzyme (Gibco, Thermo Fisher Scientific, MA, USA) and centrifuged at 400 g for 4 min at 4 °C to wash cells with ice-cold binding buffer twice (HBSS (Gibco, Thermo Fisher Scientific, MA, USA) supplemented with 20 mM HEPES and 0.5% (w/v) BSA, pH 7.4). The cells were seeded at 100,000 cells per well in 80 μL of binding buffer in a clear 96-well U-bottom plate.

For the competition binding assay, cells were treated with 10 μL of a fixed concentration of sCy5-MT-I (3 nM for MC1R, MC3R, and MC5R, or 15 nM for MC4R), along with increasing concentrations of the compounds diluted in binding buffer (10 μL, 1:10 dilution, ranging from 1 μM to 1 μM). After incubation for 2 h at 4 °C (protected from light and placed on ice), the cells were washed twice with 90 μL of ice-cold binding buffer, and the cell pellet was resuspended in 100 μL of ice-cold binding buffer containing propidium iodide (PI) solution (1:2000 dilution).

Each sample was analyzed using a Stratedigm S1000EXI flow cytometer (Stratedigm, CA, USA) with excitation at 642 nm and emission between 661 and 690 nm for sCy5 detection, and excitation at 488 nm with emission between 600 and 630 nm for PI detection. Data were processed using Cell Capture analysis software (Stratedigm, CA, USA) and further analyzed using FlowJo software (Flowjo, Inc., OR, USA) and GraphPad (Prism 10). The experiment was repeated twice, with three replicates in each experiment.

### NMR Conformational Analysis

NMR samples were prepared by dissolving the selected peptides in 0.54 mL H_2_O and 0.06 mL of ^2^H_2_O (pH 5.5) to obtain a final concentration of 1 mM. NMR spectra were recorded on a Bruker Avance NEO 600 MHz spectrometer equipped with a z-gradient 5 mm triple-resonance probe head at 25 °C. One-dimensional (1D) NMR spectra were recorded in Fourier mode with quadrature detection. 2D DQF-COSY,^38,39^ TOCSY,^40^ and NOESY^41^ spectra were recorded in the phase-sensitive mode using the method from States.^42^ Qualitative and quantitative analyses of the 2D NMR spectra were performed using the program XEASY.^43^ Complete ^1^H NMR chemical shift assignments were achieved for the main conformers of **FM635** and **FM636** according to the Wüthrich procedure^44^ with the support of the XEASY software package (Supporting Information, Tables S2 and S3). NOE-based distance restraints were obtained from NOESY spectra acquired with a mixing time of 100 ms (Supporting Information, Tables S4 and S5) and used as constraints in the simulated annealing calculations using the program CYANA.^45^ A modified protocol for determining multiple-state ensembles of proteins developed by Vogeli et al. was used to consider the intrinsic flexibility of the peptides.^34^ In particular, a two-state ensemble calculation was performed considering the NOE-derived constraints only as upper distance limits. The obtained 100 conformers were clustered using Molmol considering the heavy atoms of the pharmacophore residues (6–9) and the best 10 structures of the most populated cluster (30/100 and 33/100 structures, respectively) were optimized using the Discover algorithm (Accelrys, San Diego, CA) and the consistent valence force field.^46^ Minimization lowered the total energy of the structures, and no residue was found in the disallowed region of the Ramachandran plot. Molecular graphic images were obtained using the UCSF Chimera package.^47^

### Molecular Docking

Initial structures of *h*MC3R and *h*MC4R were generated using AlphaFold3.^48^ The *N*-terminal regions, corresponding to residues 1–34 in *h*MC3R and 1–39 in *h*MC4R, were excluded from the receptor models used for docking because of their low predicted reliability and because these regions are not resolved in the available experimental structures of melanocortin receptor–peptide complexes. Peptide **FM635** was initially manually docked into both receptors by using available crystal structures of *h*MC3R/*h*MC4R-peptide complexes as structural templates. In particular, the side chains of the pharmacophore residues 7–9 were superimposed onto the corresponding residues of the cocrystallized peptide ligands to generate the initial binding poses. Ten starting poses were generated for each receptor–ligand complex. The resulting complexes were refined by in vacuo energy minimization using the Discover algorithm. Minimization was performed with the steepest descent method followed by conjugate gradient minimization until an energy gradient convergence criterion of 0.05 kcal mol^–1^ Å^–1^ was reached. The best-scored pose for each receptor was selected for analysis and is shown in Figure 5.

## Supplementary Material









## Notes

Bar-diagram to highlight the selectivity of synthesized compounds (PDB)
Figure S18. Coordinate files of models of the complexes *h*MC3R**/FM635** and *h*MC4R**/FM635** (PDB)
Molecular formula strings (CSV)
Table S1. Analytical data of peptides **FM635**–**FM648**. Figures S1–S14. HPLC traces of the synthesized peptides. Figure S15. MS spectra of the synthesized peptides. Tables S2–S3. NMR Resonance Assignments of **FM635** and **FM636** Tables S4–S5. NMR-derived upper limit constraints of **FM635** and **FM636** Table S6. Energy of interaction in the MCRs/**FM635** complexes Figure S16–S17. Structures of known melanocortin peptides bound to different melanocortin receptors (PDF)

## Abbreviations

1D: one-dimensional
2D: two-dimensional
COMU: 1-cyano-2-ethoxy-2-oxoethylidenaminooxy dimethylamino-morpholino-carbenium hexafluorophosphate D(2′)­Nal 2-naphtylalanine
D­(2′)­Nal: 2-naphtylalanine
Dab: 2,4-diaminobutyric acid
Dap: 2,3-diaminopropionic acid
DIEA: N,N-diisopropylethylamine
hMCR: human melanocortin receptor
NDMBA: N,N′-dimethylbarbituric acid
POMC: proopiomelanocortin
Oxyma: ethylcyano­(hydroxyimino)­acetate
PyAOP: 7-azabenzotriazol-1-yloxy trispyrrolidino-phosphoniumhexafluorophosphate.
RP-HPLC: reversed-phase high performance liquid chromatography.
TIS: triisopropylsilane.
