# Supplementary material for: Goldilocks-Inspired Design of Mid-Size Macrocycles for Selective Targeting of Human Melanocortin Receptors
Source: J Med Chem. 2026 Jun 24;69(15):18800–12. doi: 10.1021/acs.jmedchem.6c01074 (PMC13492293; doi:10.1021/acs.jmedchem.6c01074)
Supplement: Supplementary file 1 [file jm6c01074_si_004.pdf]

Supporting Information

Goldilocks-Inspired Design of Mid-Size Macrocycles for Selective Targeting of Human Melanocortin Receptors

Francesco Merlino,[a,#] Li Jia,[b,#] Ida Boccino,[a] Alfonso Carotenuto,[a] Francesco Tammaro,[a] Federica Santoro,[a] Philip E. Thompson,[b*] Paolo Grieco [a*]

[a]*Department of Pharmacy, University of Naples Federico II, via Domenico Montesano 49, 80131, Naples, Italy*
[b]*Medicinal Chemistry. Monash Institute of Pharmaceutical Sciences, Faculty of Pharmacy and Pharmaceutical Sciences, Monash University (Parkville Campus, 381 Royal Parade, Parkville VIC 3052 Australia*

* Corresponding Authors: Email: paolo.grieco@unina.it; philip.thompson@monash.edu

Table of contents

1. Analytical data of peptides **FM-635 – FM-648** (Table S1)	S2
2. HPLC chromatograms of peptides **FM-635 – FM-648** (Figures S1-14)	S3
3. MS spectra of peptides **FM-635 – FM-648** (Figure S15)	S10
4. NMR Resonance Assignments[a] of **FM635** in water solution at 5 °C (Table S2)	S12
5. NMR Resonance Assignments[a] of **FM636** in water solution at 5 °C (Table S3)	S13
6. NMR-derived upper limit constraints (Å) used in **FM635** structure calculations (Table 4)	S14
7. NMR-derived upper limit constraints (Å) used in **FM636** structure calculations (Table 5)	S15
8. Lowest energy pose DISCOVER refinement outputs (Table S6)	S16
9. Structures of DPhe7 containing peptides bound to various melanocortin receptors (Figure S16)	S17
10. Structures of D(2')Nal7 containing peptides bound to various melanocortin receptors (Figure S17)	S17
11. A bar chart illustrating the selectivity between agonist and antagonist activities of the synthesized compounds (Figure S18)	S18

Table S1. Analytical data of MSH cyclic peptide analogues.

ID	t_R (min)	Molecular Formula	$[M+H]^+_{calcd}$	$[(M+nH)/n]^+_{obs}$
**FM-635**	11.0	$C_{39}H_{57}N_{12}O_7^+$	801.4	801.6, n=1<br>401.5 n=2
**FM-636**	11.9	$C_{39}H_{57}N_{12}O_7^+$	851.4	851.6, n=1<br>426.5, n=2
**FM-639**	12.5	$C_{93}H_{52}N_{11}O_7^+$	786.4	786.6, n=1
**FM-640**	13.2	$C_{43}H_{54}N_{15}O_7^+$	836.4	836.6, n=1
**FM-637**	11.0	$C_{38}H_{51}N_{12}O_7^+$	787.4	787.5, n=1<br>394.5, n=2
**FM-638**	11.3	$C_{42}H_{53}N_{12}O_7^+$	837.4	837.6 n=1<br>419.5, n=2
**FM-641**	11.9	$C_{38}H_{50}N_{11}O_7^+$	772.4	772.6, n=1
**FM-642**	12.4	$C_{42}H_{52}N_{11}O_7^+$	822.4	822.6, n=1
**FM-643**	10.9	$C_{46}H_{59}N_{14}O_8^+$	935.5	935.6, n=1<br>468.6, n=2
**FM-644**	11.3	$C_{50}H_{60}N_{14}O_8^+$	985.5	985.7, n=1<br>493.6, n=2
**FM-645**	10.1	$C_{45}H_{59}N_{14}O_8^+$	923.5	923.6, n=1<br>462.5, n=2
**FM-646**	11.1	$C_{49}H_{61}N_{14}O_8^+$	973.5	973.6, n=1<br>487.6, n=2
**FM-647**	10.1	$C_{45}H_{59}N_{14}O_8^+$	923.5	923.6, n=1<br>462.5, n=2
**FM-648**	11.1	$C_{49}H_{61}N_{14}O_8^+$	973.5	973.6, n=1<br>487.6, n=2

HPLC chromatograms of peptides investigated in this study.

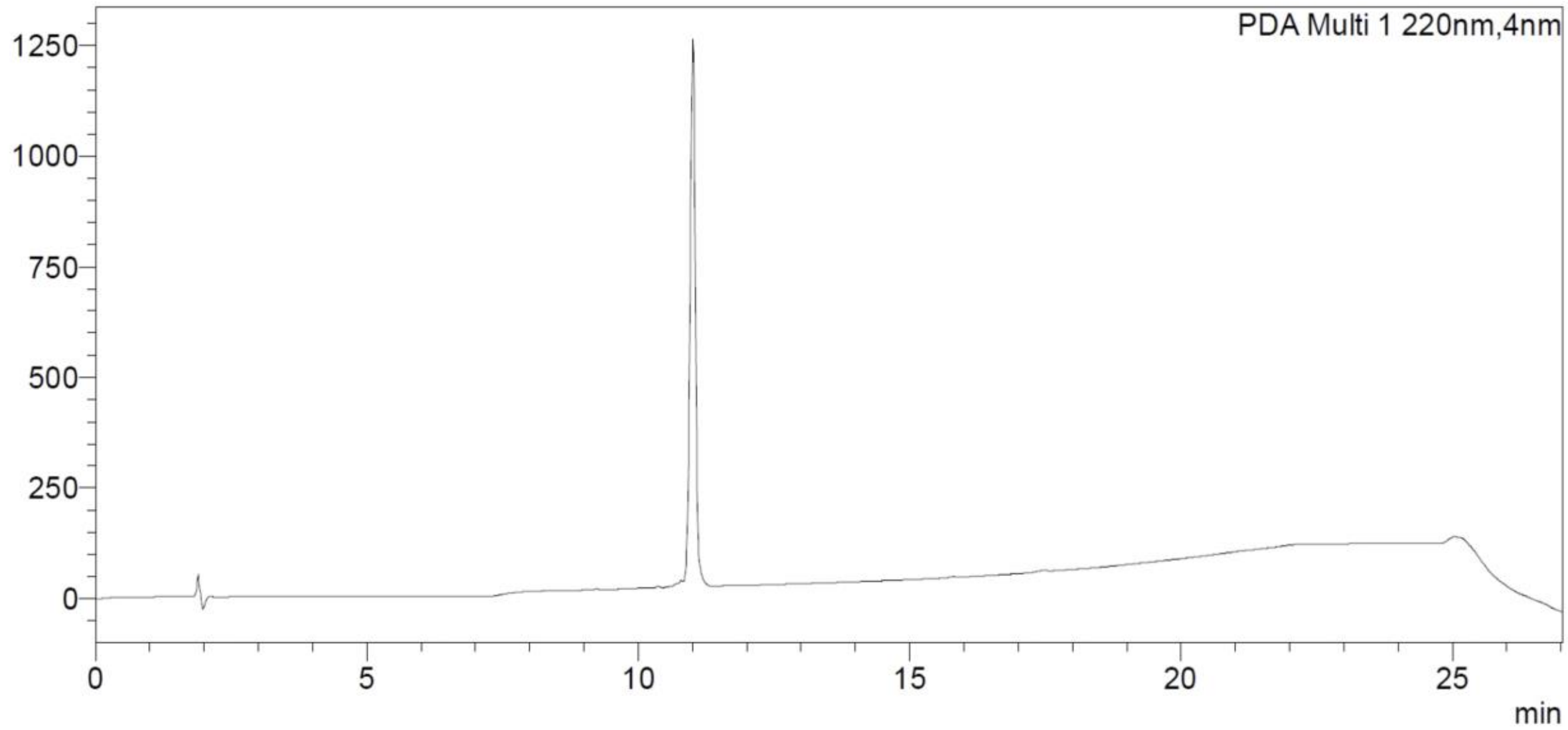

Figure S1. Chromatogram of **FM-635** obtained by an analytical HPLC (Shimadzu UFLC SPD-M20A/DGU-20A3R/LC-20AD) equipped with a Phenomenex Kinetex C18 column (150 mm × 4.6 mm, 5 µm, 100 Å), and monitored by UV detection at 220 nm. t_R= 11.0 min [linear gradient 10-90% MeCN (0.1% TFA) in H_2O (0.1% TFA) over 15 min, flow rate of 1 mL/min].

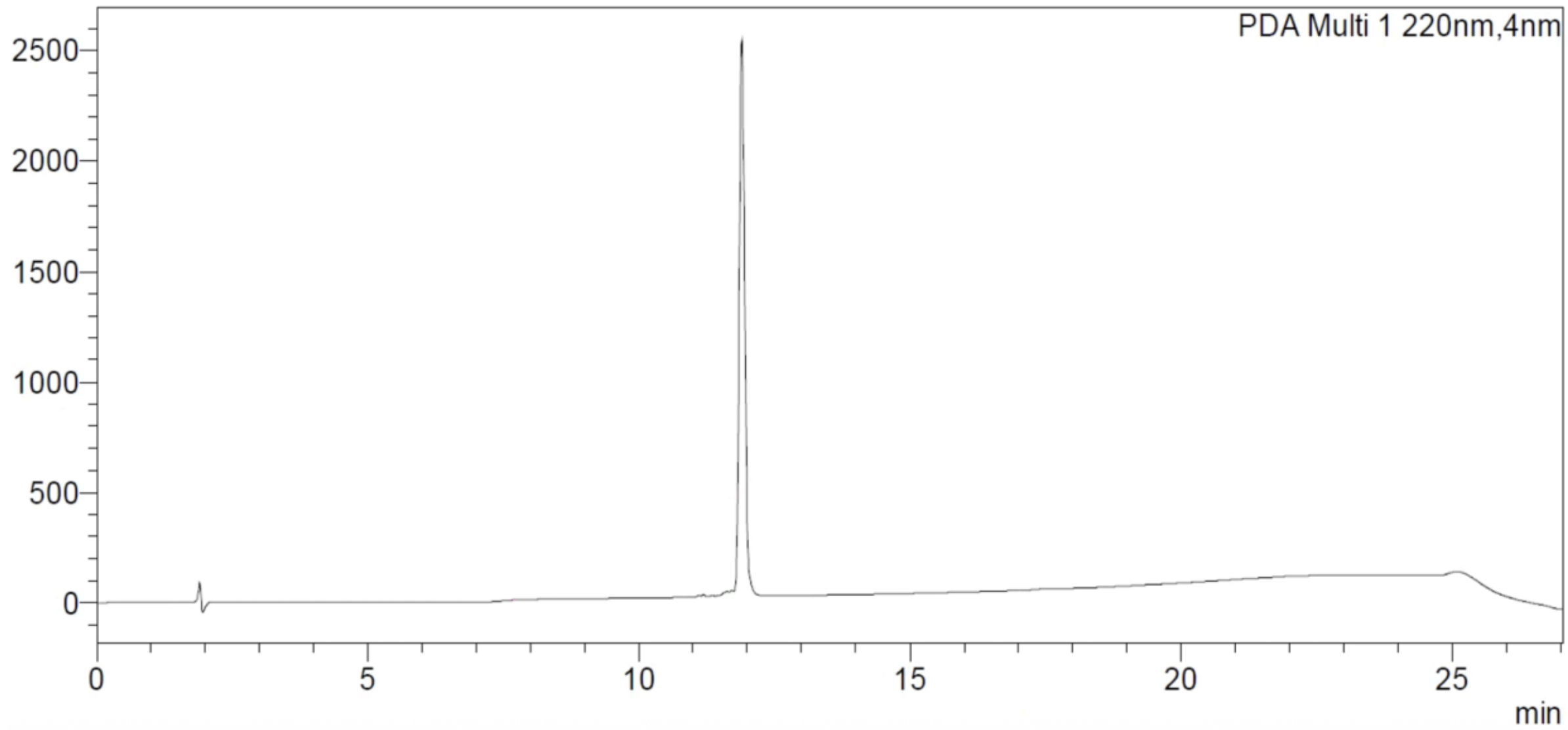

Figure S2. Chromatogram of **FM-636** obtained by an analytical HPLC (Shimadzu UFLC SPD-M20A/DGU-20A3R/LC-20AD) equipped with a Phenomenex Kinetex C18 column (150 mm × 4.6 mm, 5 µm, 100 Å), and monitored by UV detection at 220 nm. t_R= 11.9 min [linear gradient 10-90% MeCN (0.1% TFA) in H_2O (0.1% TFA) over 15 min, flow rate of 1 mL/min].

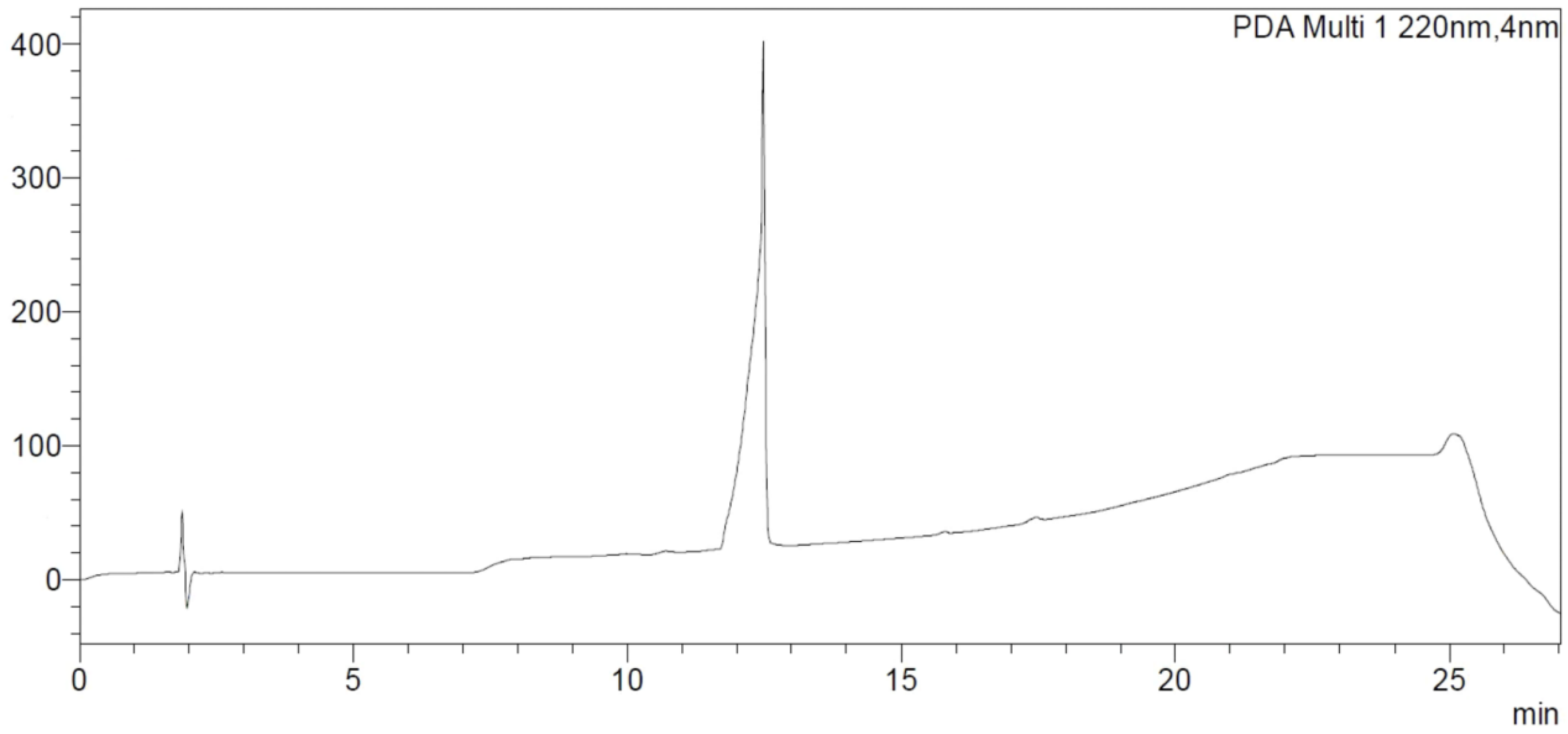

Figure S3. Chromatogram of **FM-639** obtained by an analytical HPLC (Shimadzu UFLC SPD-M20A/DGU-20A3R/LC-20AD) equipped with a Phenomenex Kinetex C18 column (150 mm × 4.6 mm, 5 µm, 100 Å), and monitored by UV detection at 220 nm. t_R= 12.5 min [linear gradient 10-90% MeCN (0.1% TFA) in H_2O (0.1% TFA) over 15 min, flow rate of 1 mL/min].

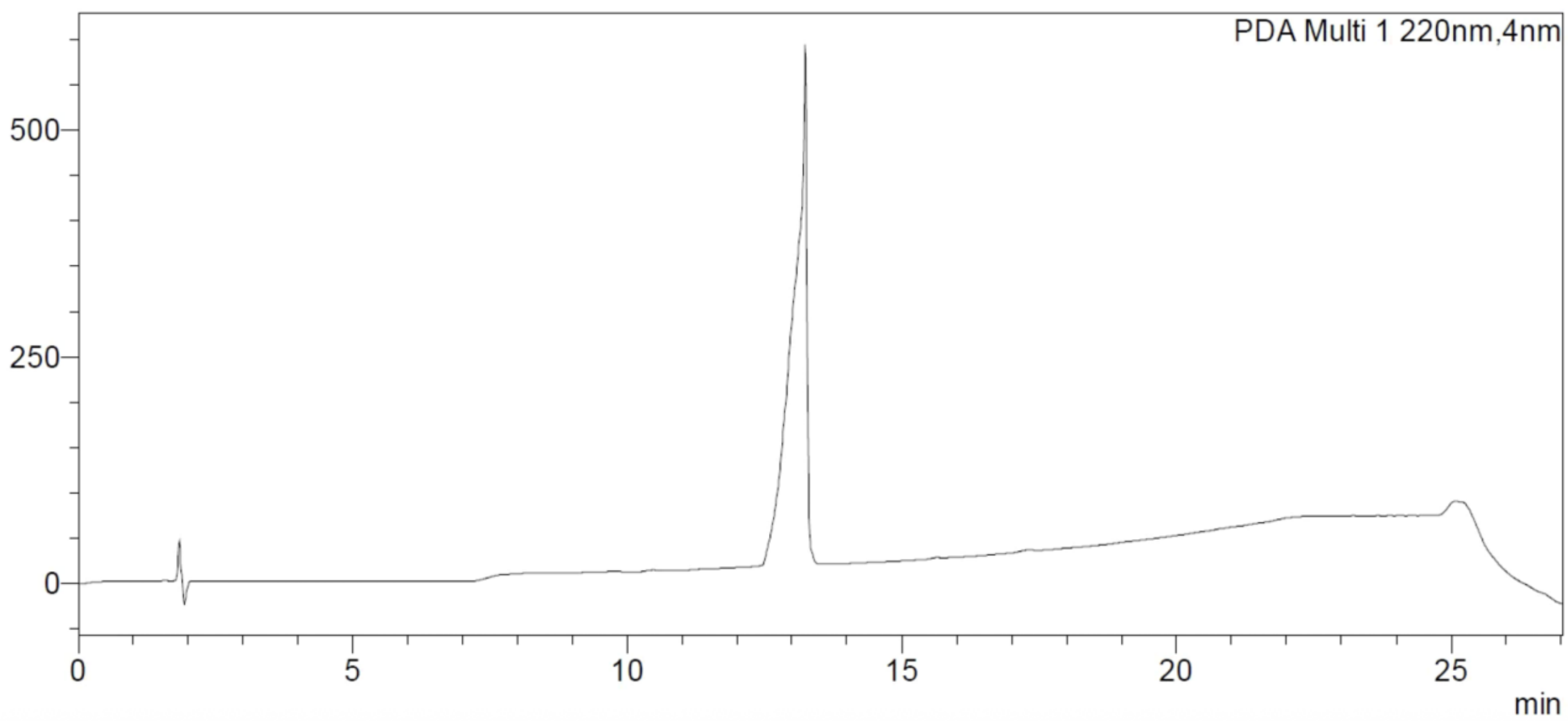

Figure S4. Chromatogram of **FM-640** obtained by an analytical HPLC (Shimadzu UFLC SPD-M20A/DGU-20A3R/LC-20AD) equipped with a Phenomenex Kinetex C18 column (150 mm × 4.6 mm, 5 µm, 100 Å), and monitored by UV detection at 220 nm. t_R= 13.2 min [linear gradient 10-90% MeCN (0.1% TFA) in H_2O (0.1% TFA) over 15 min, flow rate of 1 mL/min].

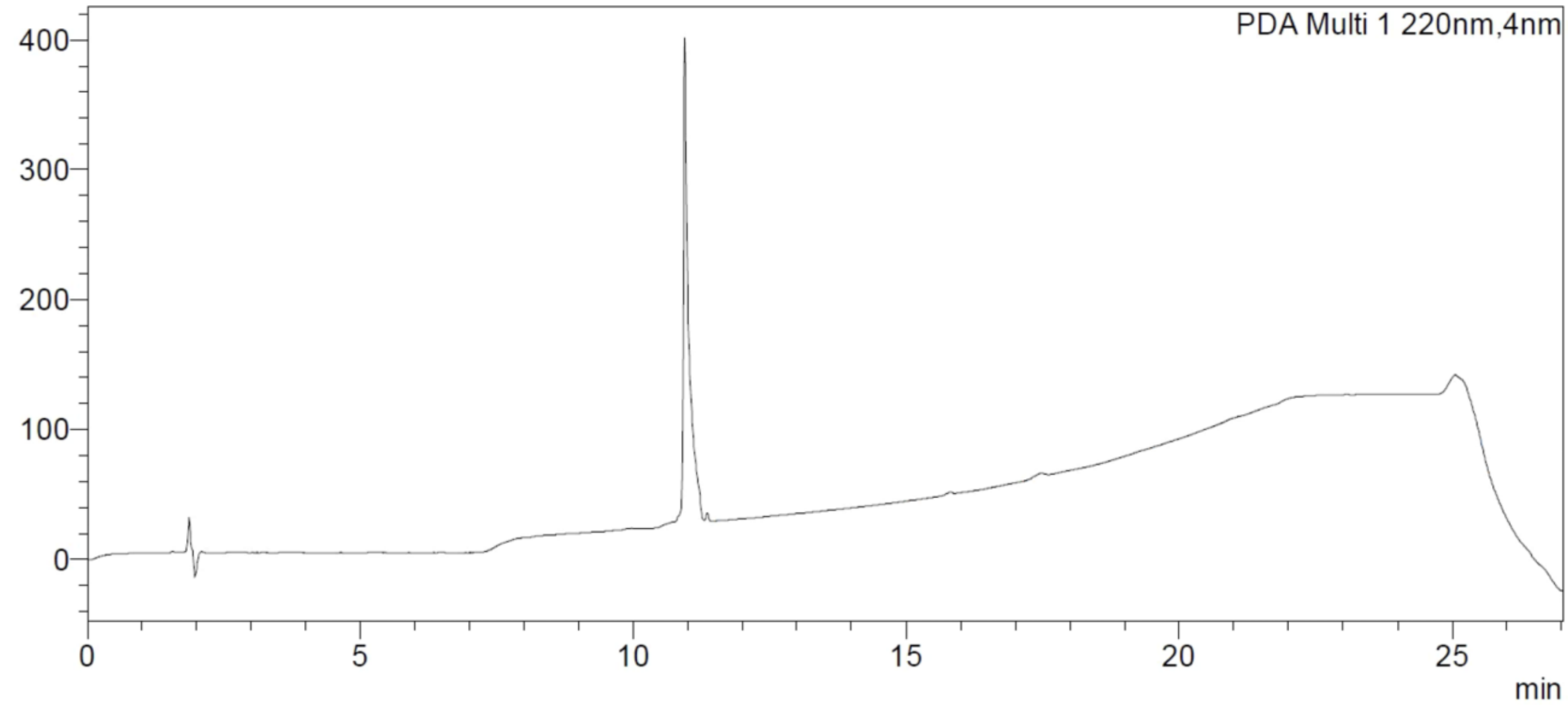

Figure S5. Chromatogram of **FM-637** obtained by an analytical HPLC (Shimadzu UFLC SPD-M20A/DGU-20A3R/LC-20AD) equipped with a Phenomenex Kinetex C18 column (150 mm × 4.6 mm, 5 µm, 100 Å), and monitored by UV detection at 220 nm. t_R= 11.0 min [linear gradient 10-90% MeCN (0.1% TFA) in H_2O (0.1% TFA) over 15 min, flow rate of 1 mL/min].

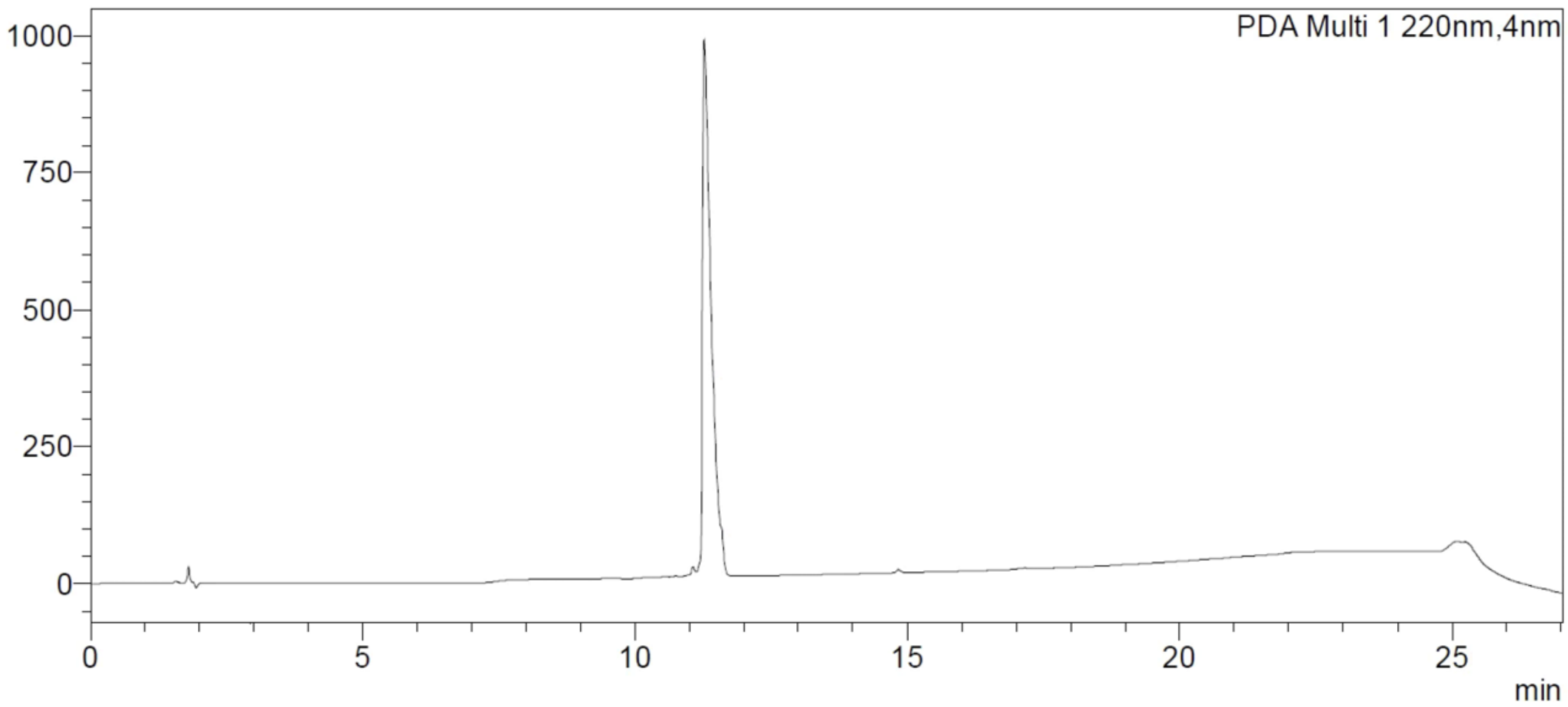

Figure S6. Chromatogram of **FM-638** obtained by an analytical HPLC (Shimadzu UFLC SPD-M20A/DGU-20A3R/LC-20AD) equipped with a Phenomenex Kinetex C18 column (150 mm × 4.6 mm, 5 µm, 100 Å), and monitored by UV detection at 220 nm. t_R= 11.3 min [linear gradient 10-90% MeCN (0.1% TFA) in H_2O (0.1% TFA) over 15 min, flow rate of 1 mL/min].

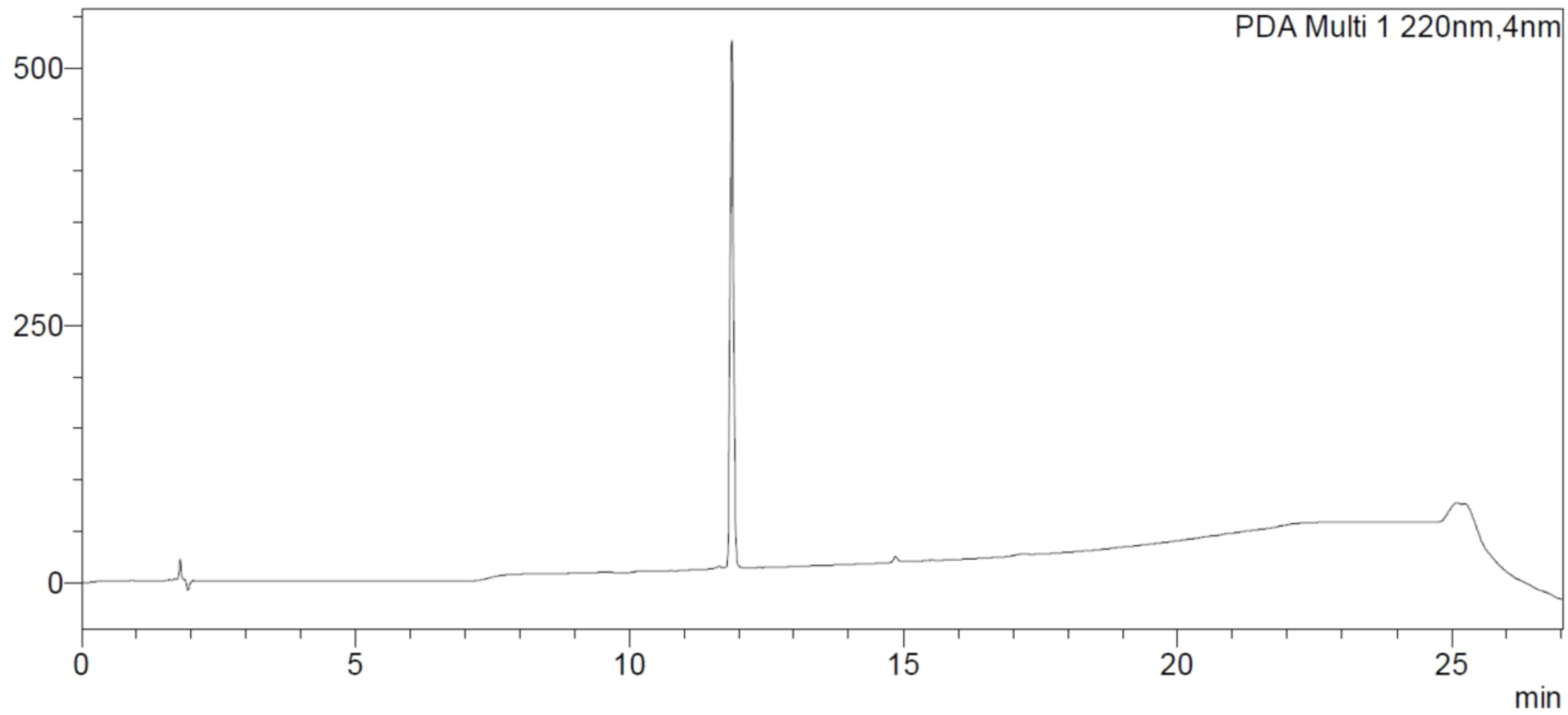

Figure S7. Chromatogram of **FM-641** obtained by an analytical HPLC (Shimadzu UFLC SPD-M20A/DGU-20A3R/LC-20AD) equipped with a Phenomenex Kinetex C18 column (150 mm × 4.6 mm, 5 µm, 100 Å), and monitored by UV detection at 220 nm. t_R= 11.9 min [linear gradient 10-90% MeCN (0.1% TFA) in H_2O (0.1% TFA) over 15 min, flow rate of 1 mL/min].

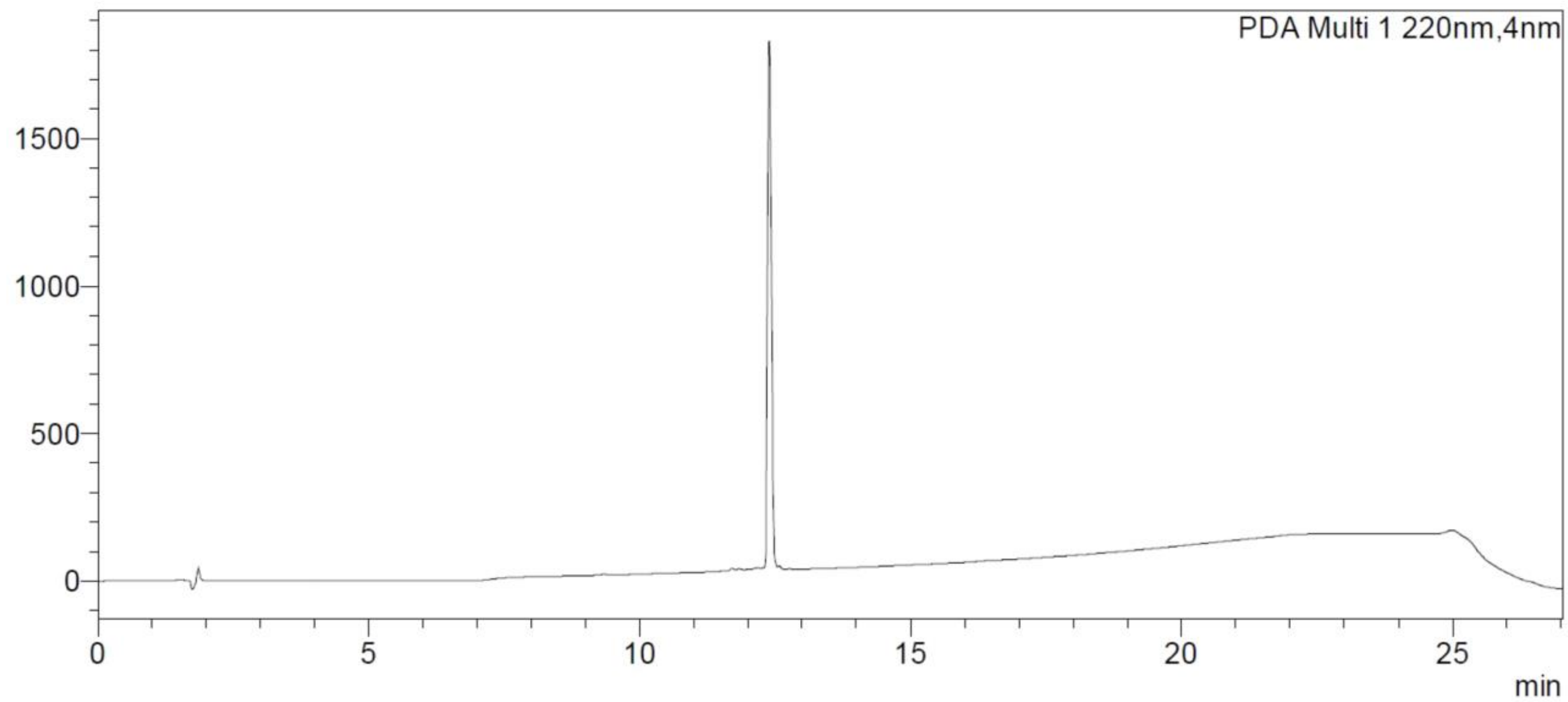

Figure S8. Chromatogram of **FM-642** obtained by an analytical HPLC (Shimadzu UFLC SPD-M20A/DGU-20A3R/LC-20AD) equipped with a Phenomenex Kinetex C18 column (150 mm × 4.6 mm, 5 µm, 100 Å), and monitored by UV detection at 220 nm. t_R= 12.4 min [linear gradient 10-90% MeCN (0.1% TFA) in H_2O (0.1% TFA) over 15 min, flow rate of 1 mL/min].

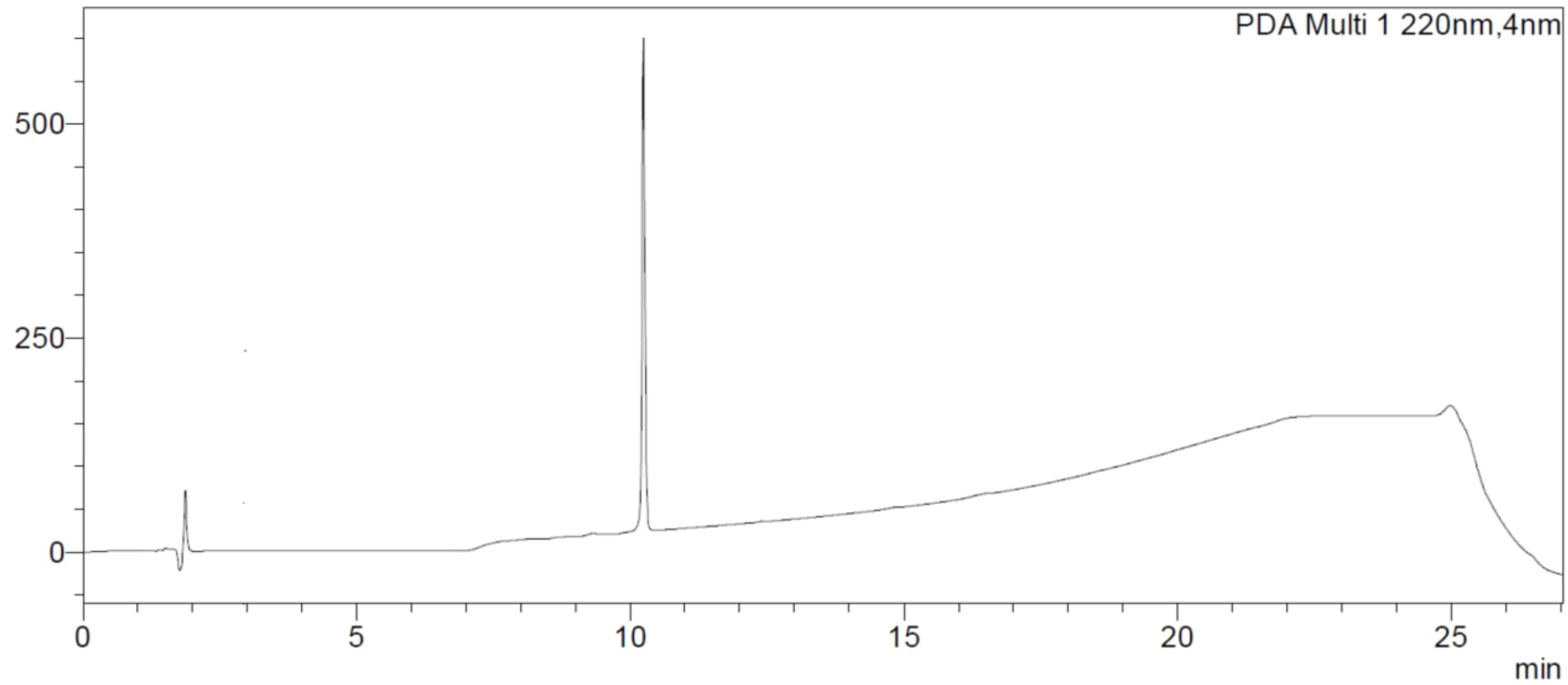

Figure S9. Chromatogram of **FM-643** obtained by an analytical HPLC (Shimadzu UFLC SPD-M20A/DGU-20A3R/LC-20AD) equipped with a Phenomenex Kinetex C18 column (150 mm × 4.6 mm, 5 µm, 100 Å), and monitored by UV detection at 220 nm. t_R= 10.9 min [linear gradient 10-90% MeCN (0.1% TFA) in H_2O (0.1% TFA) over 15 min, flow rate of 1 mL/min].

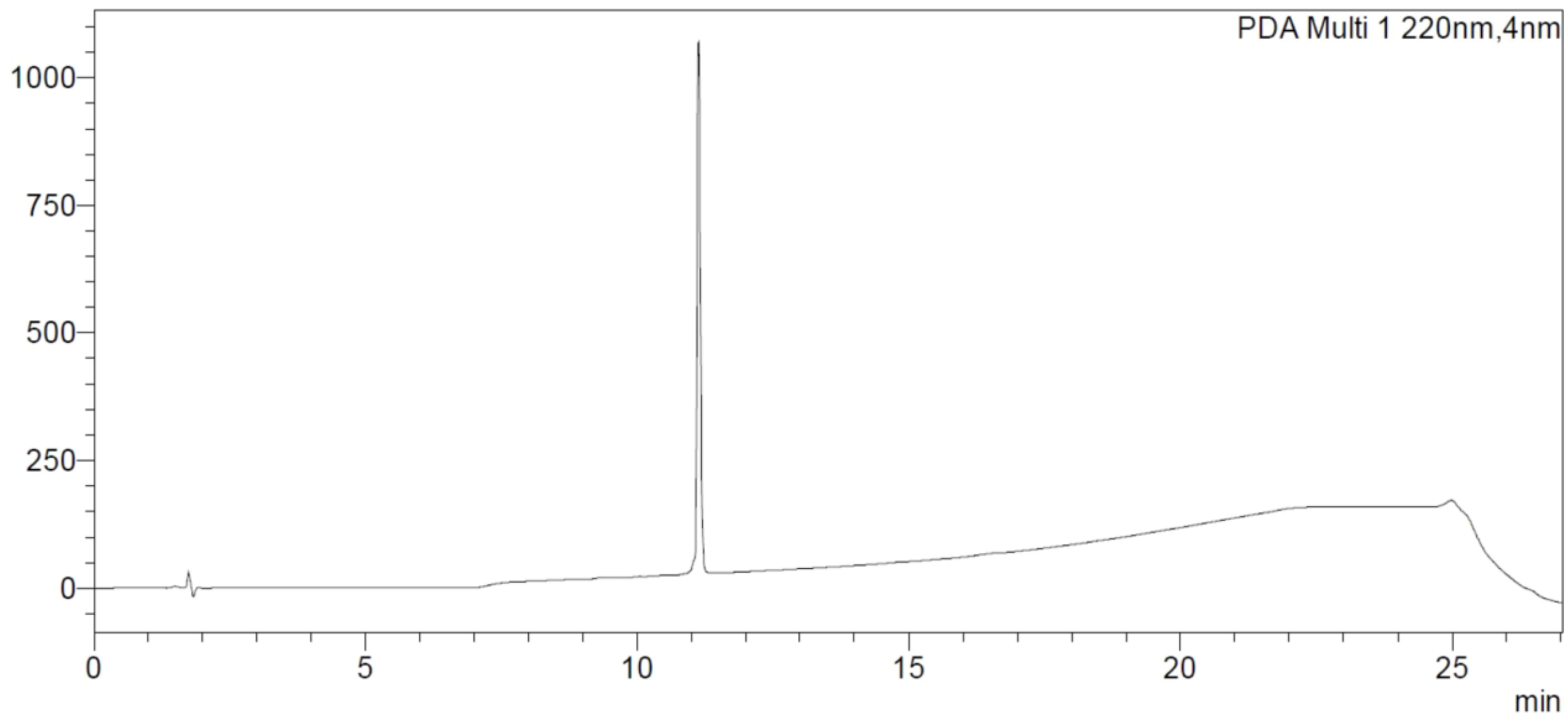

Figure S10. Chromatogram of **FM-644** obtained by an analytical HPLC (Shimadzu UFLC SPD-M20A/DGU-20A3R/LC-20AD) equipped with a Phenomenex Kinetex C18 column (150 mm × 4.6 mm, 5 µm, 100 Å), and monitored by UV detection at 220 nm. t_R= 11.3 min [linear gradient 10-90% MeCN (0.1% TFA) in H_2O (0.1% TFA) over 15 min, flow rate of 1 mL/min].

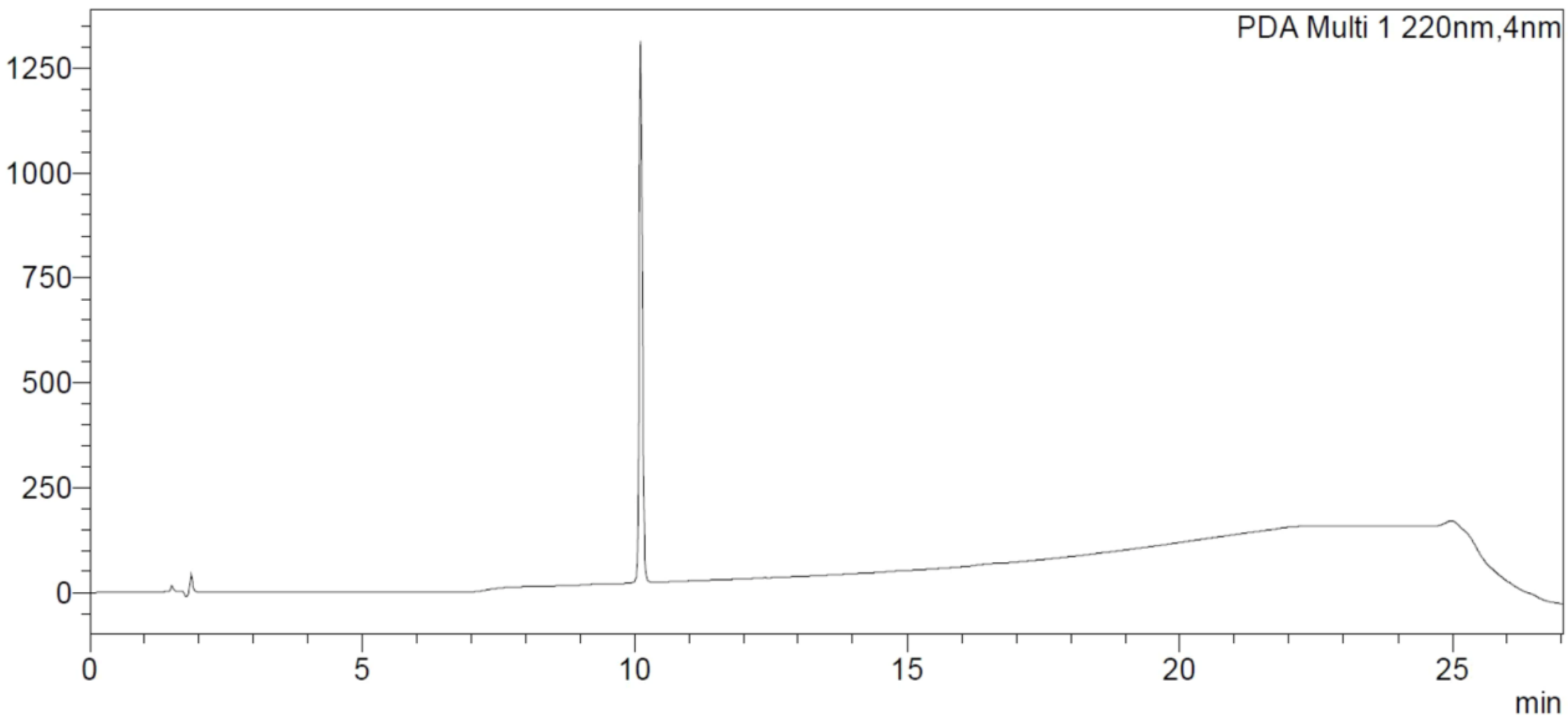

Figure S11. Chromatogram of **FM-645** obtained by an analytical HPLC (Shimadzu UFLC SPD-M20A/DGU-20A3R/LC-20AD) equipped with a Phenomenex Kinetex C18 column (150 mm × 4.6 mm, 5 µm, 100 Å), and monitored by UV detection at 220 nm. t_R= 10.1 min [linear gradient 10-90% MeCN (0.1% TFA) in H_2O (0.1% TFA) over 15 min, flow rate of 1 mL/min].

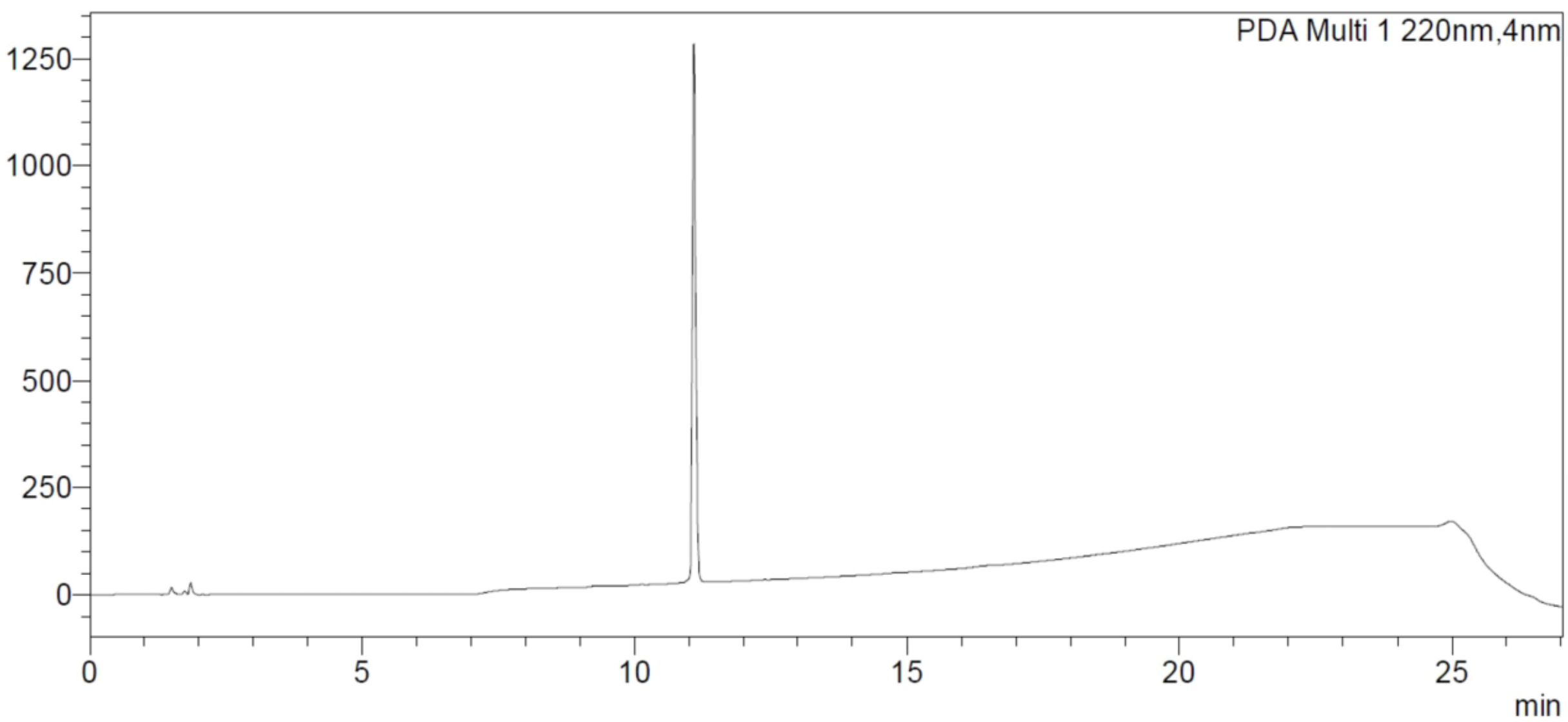

Figure S12. Chromatogram of **FM-646** obtained by an analytical HPLC (Shimadzu UFLC SPD-M20A/DGU-20A3R/LC-20AD) equipped with a Phenomenex Kinetex C18 column (150 mm × 4.6 mm, 5 µm, 100 Å), and monitored by UV detection at 220 nm. t_R= 11.1 min [linear gradient 10-90% MeCN (0.1% TFA) in H_2O (0.1% TFA) over 15 min, flow rate of 1 mL/min].

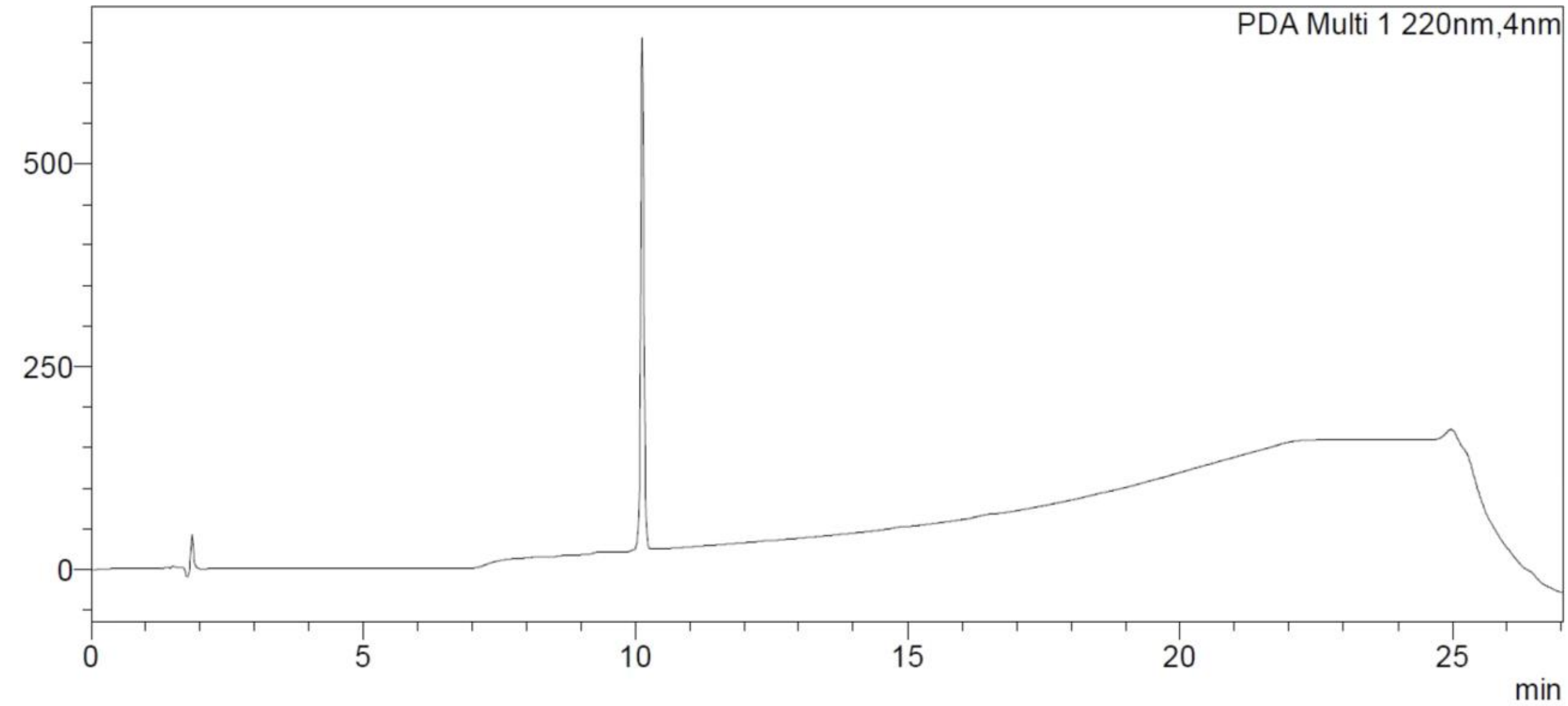

Figure S13. Chromatogram of **FM-647** obtained by an analytical HPLC (Shimadzu UFLC SPD-M20A/DGU-20A3R/LC-20AD) equipped with a Phenomenex Kinetex C18 column (150 mm × 4.6 mm, 5 µm, 100 Å), and monitored by UV detection at 220 nm. t_R= 10.1 min [linear gradient 10-90% MeCN (0.1% TFA) in H_2O (0.1% TFA) over 15 min, flow rate of 1 mL/min].

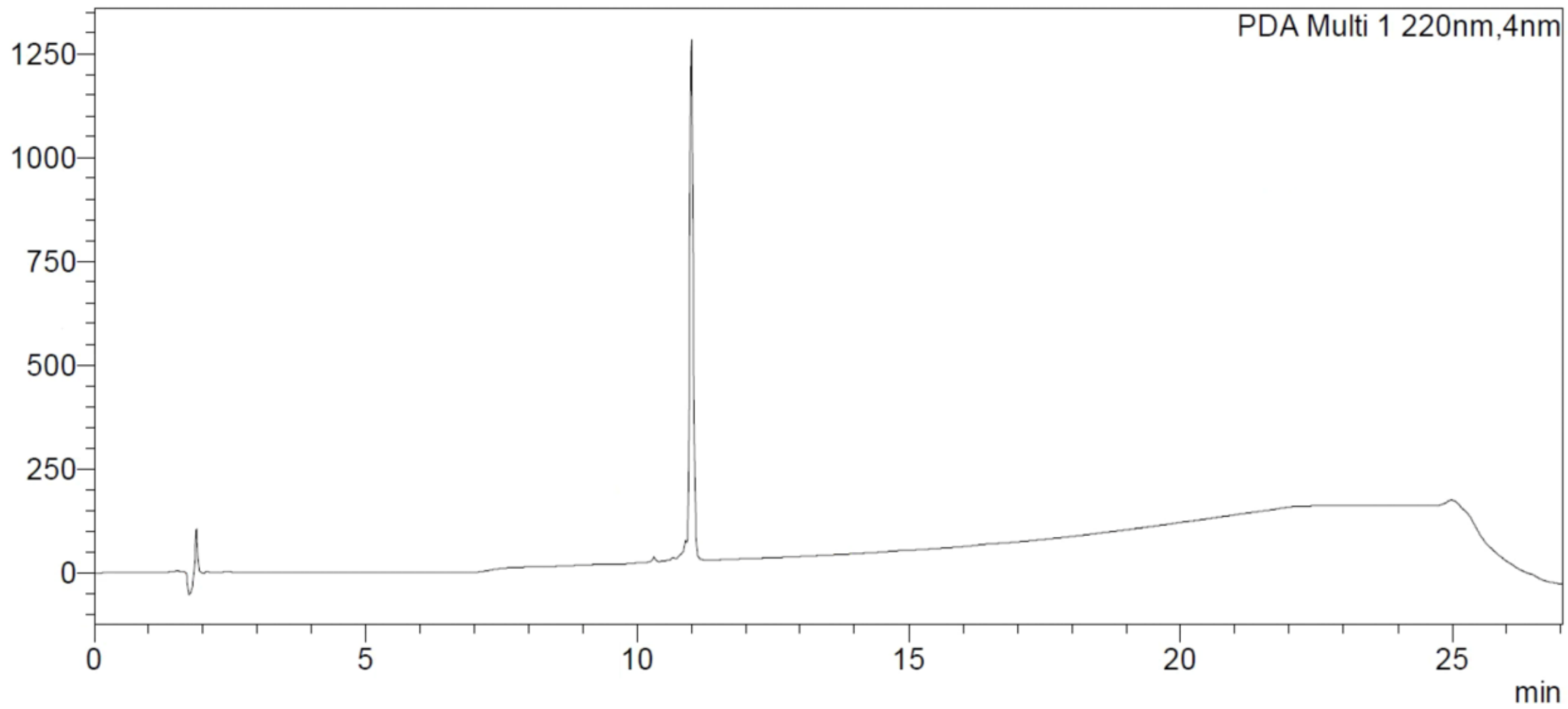

Figure S14. Chromatogram of **FM-648** obtained by an analytical HPLC (Shimadzu UFLC SPD-M20A/DGU-20A3R/LC-20AD) equipped with a Phenomenex Kinetex C18 column (150 mm × 4.6 mm, 5 µm, 100 Å), and monitored by UV detection at 220 nm. t_R= 11.1 min [linear gradient 10-90% MeCN (0.1% TFA) in H_2O (0.1% TFA) over 15 min, flow rate of 1 mL/min].

*MS spectra of MSH cyclic peptides (**FM635-FM648**).*

(A)

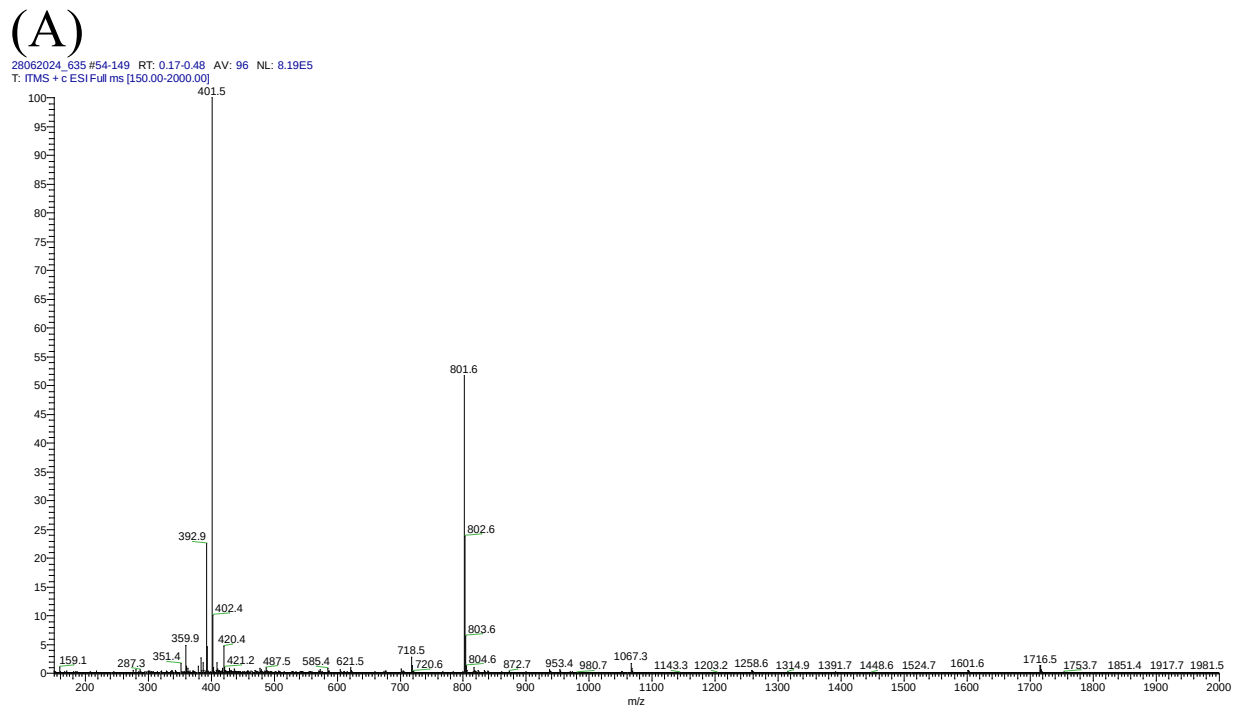

(B)

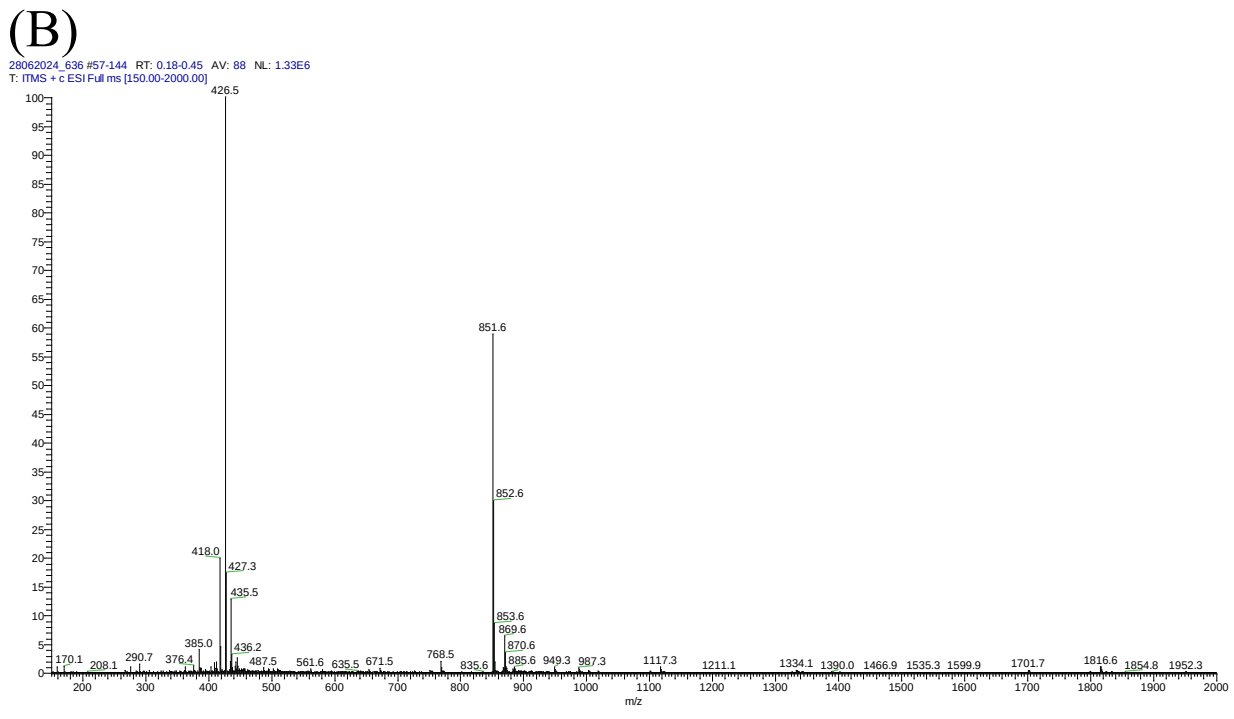

(C)

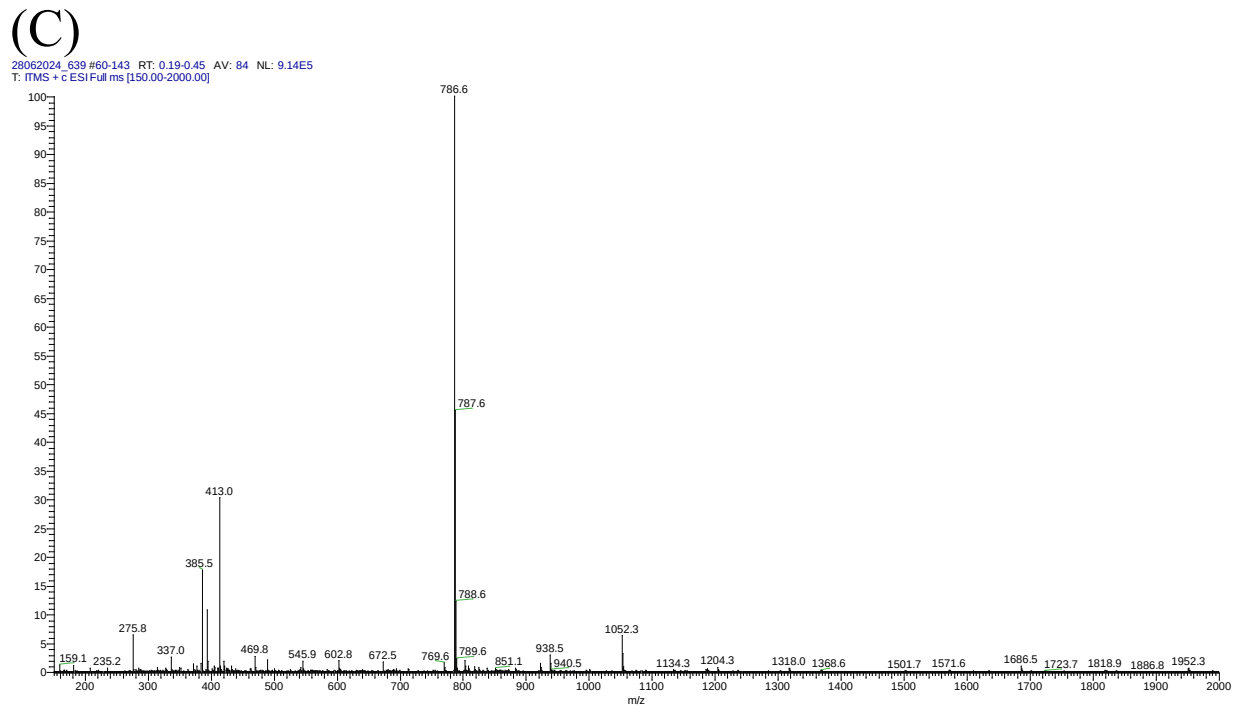

(D)

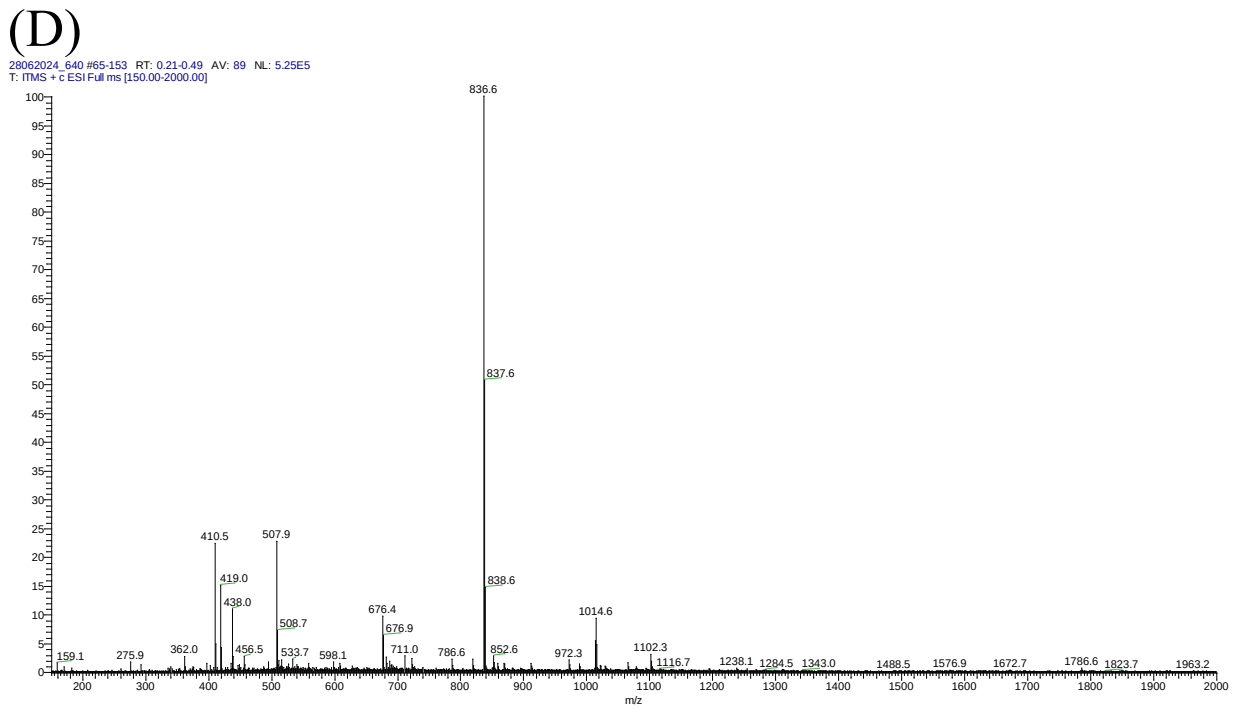

(E)

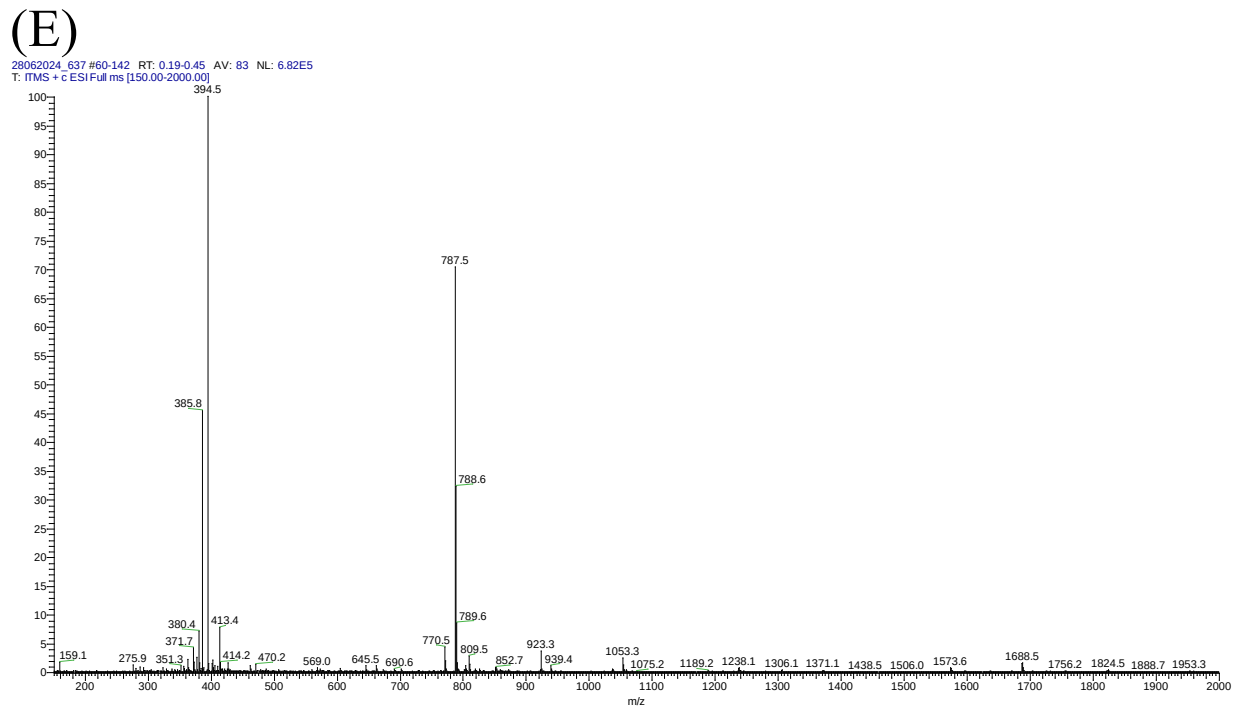

(F)

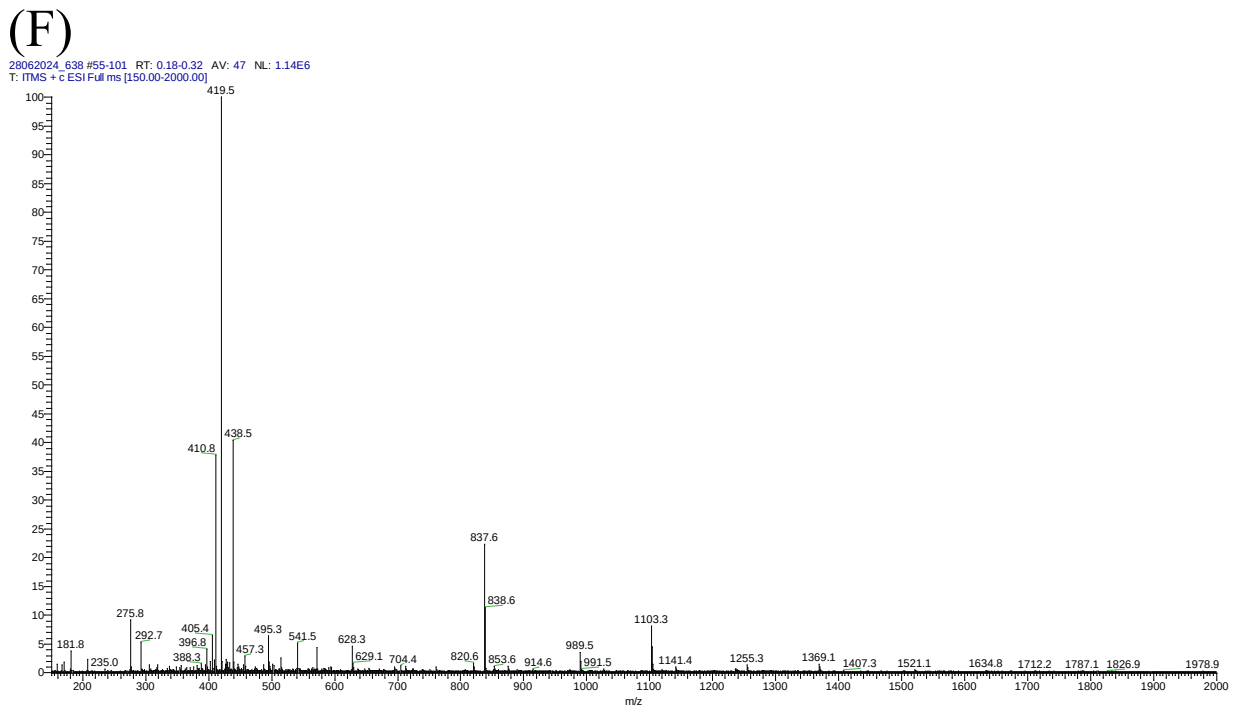

(G)

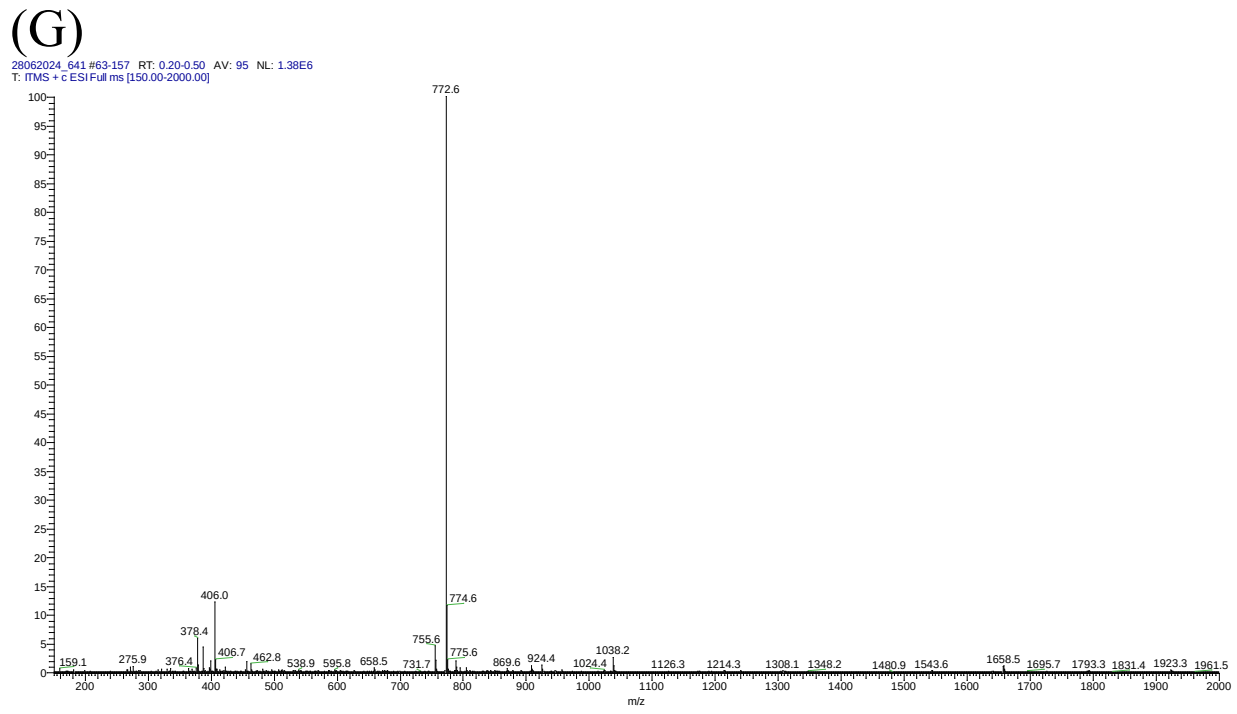

(H)

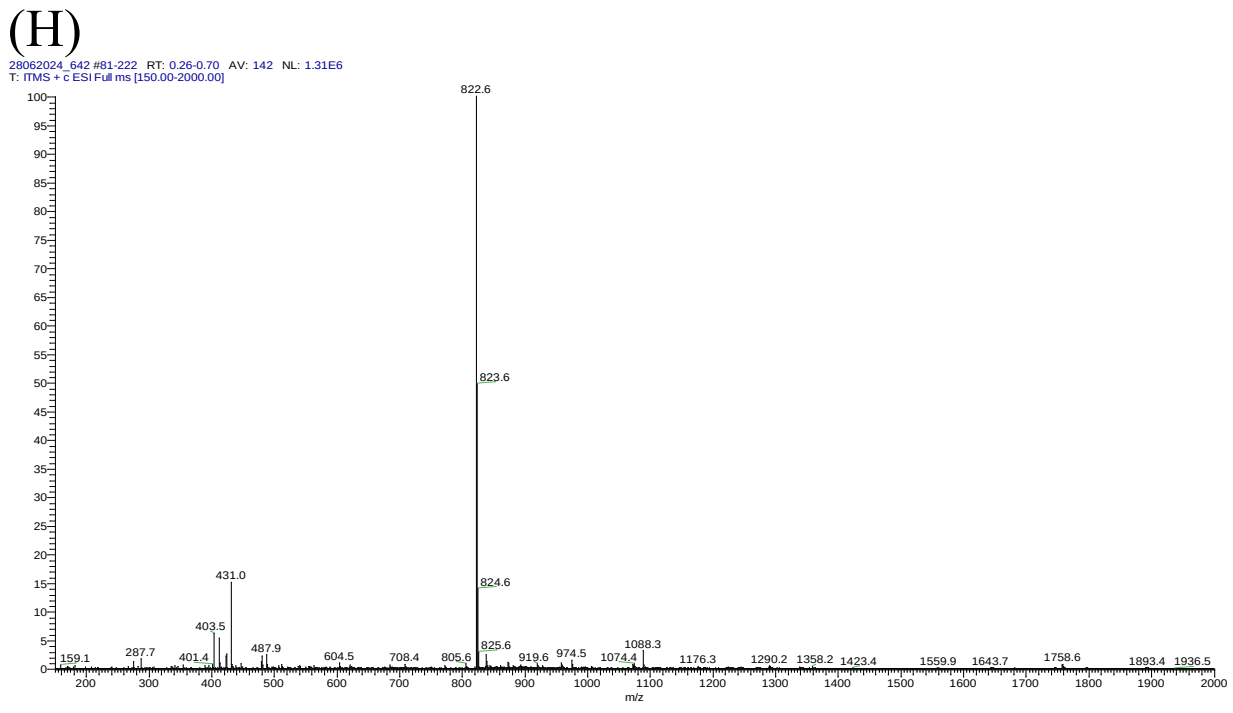

(I)

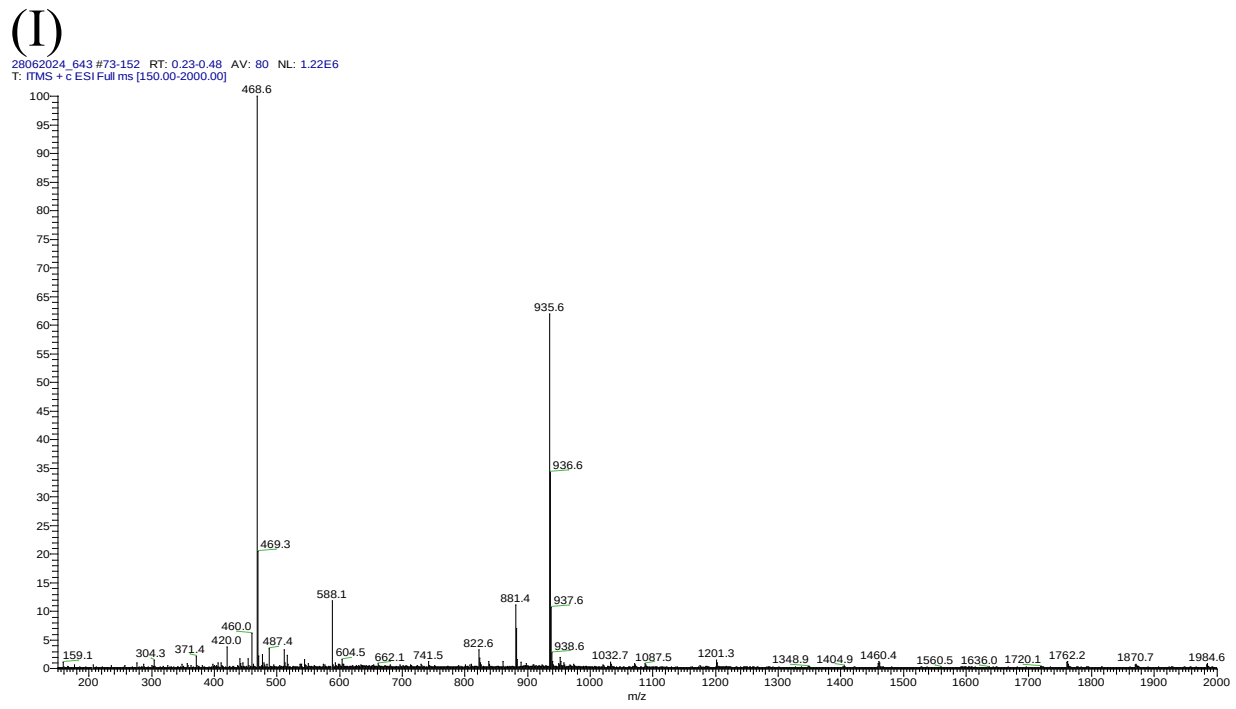

(J)

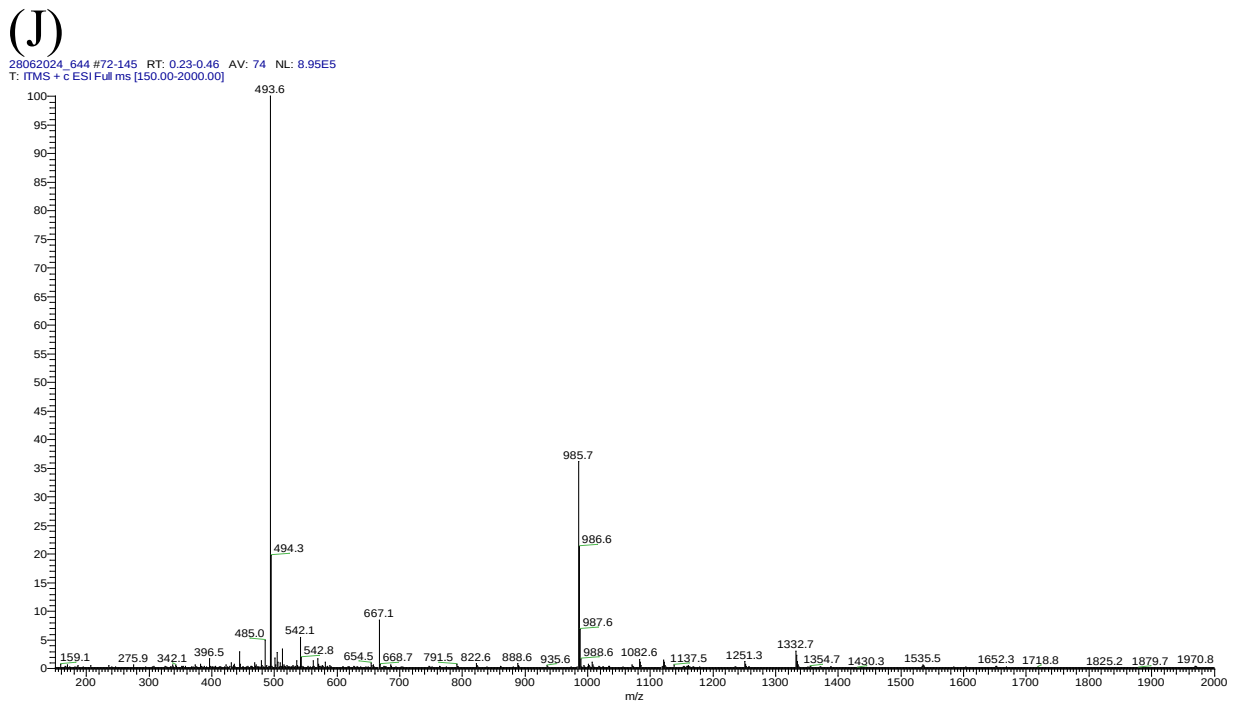

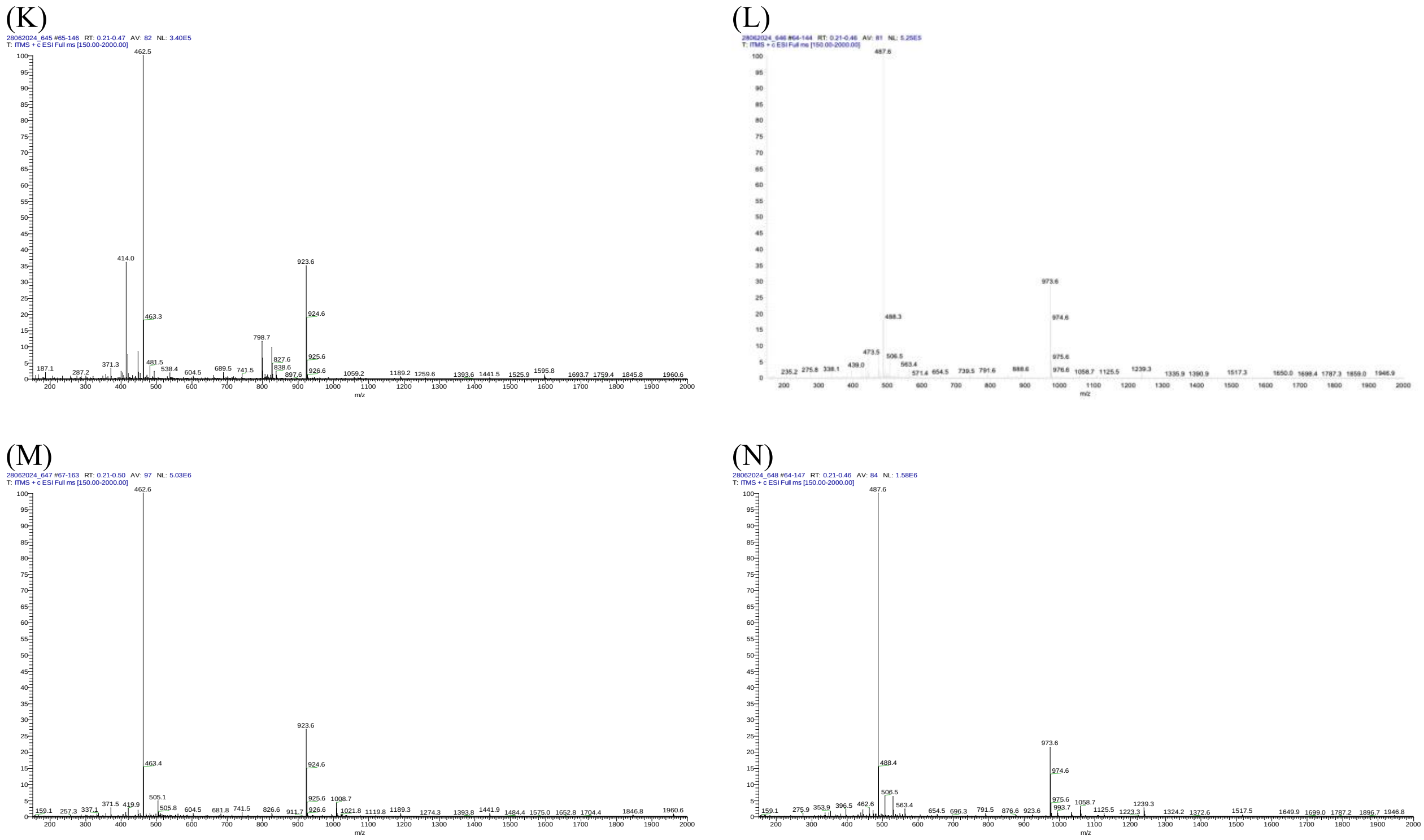

Figure S15. MS spectra for **FM-635** (A), **FM-636** (B), **FM-639** (C), **FM-640** (D), **FM-637** (E), **FM-638** (F), **FM-641**(G), **FM-642** (H), **FM-643** (I), **FM-644** (J), **FM-645** (K), **FM-646** (L), **FM-647** (M), **FM-648** (N).

Table S2. NMR Resonance Assignments[a] of **FM635** in water solution at 5 °C.

Residue	NH ($^3J_{\alpha N}$, -Δδ/ΔT)[b]	$C^{\alpha}H$	$C^{\beta}H$	Others
Dab[5]		4.43	2.14; 2.06;	3.42, 3.12(γ); 7.89(δ)
Pro[6]		4.58	1.69; 2.13	1.99(γ); 3.60, 3.68(δ);
DPhe[7]	8.74 (6.4, 10.5)	4.55	3.09	7.27(δ); 7.36(ε); 7.29 (ζ)
Arg[8]	8.05 (7.4, 9.0)	4.10	1.40; 1.23	0.88, 0.80(γ); 2.84(δ); 7.01(ε)
Trp[9]	8.37 (4.9, 8.7)	4.63	3.30	7.26(δ); 7.62, 10.23(ε); 7.16, 7.47, (ζ); 7.25(η)
Asn[10]	8.12 (7.2, 1.4)	4.20	2.48; 2.28	7.57, 6.84(δ);

[a]Obtained at pH = 5.4, with TSP (δ 0.00 ppm) as reference shift. Chemical shifts are accurate to ±0.02 ppm.

[b] $^3J_{\alpha N}$ coupling constants in Hz.

-Δδ/ΔT = temperature coefficients (ppb/K) calculated in the range 5-25 °C.

Table S3. NMR Resonance Assignments[a] of **FM636** in water solution at 5 °C.

Residue	NH ($^{3}J_{\alpha N}$, -Δδ/ΔT)[b]	C$^{\alpha}$H	C$^{\beta}$H	Others
Dab[5]		4.42	2.12; 2.03;	3.41, 3.18(γ); 7.94(δ)
Pro[6]		4.62	1.84; 2.17	2.01(γ); 3.63, 3.69(δ);
DBNA[7]	8.82 (5.8, 10.2)	4.59	3.32; 3.19	7.45, 7.71(δ); 7.92, 7.88, 7.55(ε); 7.90, 7.54(ζ)
Arg[8]	7.91 (7.5, 9.0)	3.94	0.98, 0.80	0.27, 0.19(γ); 2.23, 2.11, 2.24(δ); 6.51(ε)
Trp[9]	8.21 (6.3, 6.9)	4.61	3.25;	7.21(δ); 7.60, 10.18(ε); 7.13, 7.43(ζ); 7.22(η)
Asn[10]	8.23 (7.4, 3.7)	4.22	2.54, 2.42	7.62, 6.88(δ);

[a]Obtained at pH = 5.4, with TSP (δ 0.00 ppm) as reference shift. Chemical shifts are accurate to ±0.02 ppm.

[b] $^{3}J_{\alpha N}$ coupling constants in Hz.

-Δδ/ΔT = temperature coefficients (ppb/K) calculated in the range 5-25 °C.

Table S4. NMR-derived upper limit constraints (Å) used in **FM635** structure calculations.

5	DAB	HA	5	DAB	HD	4.90
5	DAB	HA	6	PRO	HD2	2.97
5	DAB	HA	6	PRO	HD3	2.97
5	DAB	HA	6	PRO	QD	2.49
5	DAB	HD	10	ASN	HN	3.49
5	DAB	HD	10	ASN	HA	3.32
6	PRO	HA	7	DPHE	HN	2.25
6	PRO	HB2	7	DPHE	HN	3.47
6	PRO	HB3	7	DPHE	HN	3.47
7	DPHE	HN	7	DPHE	QB	3.63
7	DPHE	HA	7	DPHE	QD	5.46
7	DPHE	HA	8	ARG	HN	2.39
7	DPHE	QB	8	ARG	HN	4.61
7	DPHE	QD	8	ARG	QB	6.50
7	DPHE	QE	8	ARG	QG	4.50
8	ARG	HN	8	ARG	HB2	3.27
8	ARG	HN	8	ARG	HB3	3.27
8	ARG	HN	8	ARG	QB	2.75
8	ARG	HN	8	ARG	HG2	4.37
8	ARG	HN	8	ARG	HG3	4.37
8	ARG	HN	8	ARG	QG	3.71
8	ARG	HN	9	TRP	HN	2.82
8	ARG	HA	8	ARG	QD	5.68
8	ARG	HA	8	ARG	HE	4.90
8	ARG	HA	9	TRP	HN	2.61
8	ARG	HB2	9	TRP	HN	3.32
8	ARG	HB3	9	TRP	HN	3.32
8	ARG	QB	9	TRP	HN	2.91
8	ARG	QB	9	TRP	HH2	3.98
8	ARG	QG	9	TRP	HE1	4.76
9	TRP	HN	9	TRP	QB	3.58
9	TRP	HN	9	TRP	HD1	3.52
9	TRP	HN	10	ASN	HN	3.19
9	TRP	HA	9	TRP	HD1	3.49
9	TRP	HA	9	TRP	HE3	3.44
9	TRP	HA	10	ASN	HN	2.58
9	TRP	QB	10	ASN	HN	4.19
10	ASN	HN	10	ASN	HB2	3.41
10	ASN	HN	10	ASN	HB3	3.41

Table S5. NMR-derived upper limit constraints (Å) used in **FM636** structure calculations.

5	DAB	HD	10	ASN	HN	2.94
5	DAB	HD	10	ASN	HA	2.80
5	DAB	HA	6	PRO	HD2	3.05
5	DAB	HA	6	PRO	HD3	3.05
5	DAB	HA	6	PRO	QD	2.64
6	PRO	HA	7	DBNA	HN	2.25
6	PRO	HB2	7	DBNA	HN	3.49
6	PRO	HB3	7	DBNA	HN	3.49
7	DBNA	HN	7	DBNA	HB2	2.64
7	DBNA	HN	7	DBNA	HB3	2.64
7	DBNA	HN	8	ARG	HN	3.44
7	DBNA	HA	8	ARG	HN	2.39
7	DBNA	HB2	7	DBNA	HD2	2.86
7	DBNA	HB3	7	DBNA	HD2	2.86
7	DBNA	QB	7	DBNA	HD2	2.33
7	DBNA	HD2	8	ARG	QG	4.76
8	ARG	HN	8	ARG	HB2	3.24
8	ARG	HN	8	ARG	HB3	3.24
8	ARG	HN	8	ARG	QB	2.78
8	ARG	HN	8	ARG	HG2	4.21
8	ARG	HN	8	ARG	HG3	4.21
8	ARG	HN	8	ARG	QG	3.65
8	ARG	HN	9	TRP	HN	2.74
8	ARG	HA	8	ARG	QD	5.68
8	ARG	HA	9	TRP	HN	2.58
8	ARG	HB2	9	TRP	HN	3.10
8	ARG	HB3	9	TRP	HN	3.10
8	ARG	QB	9	TRP	HH2	4.06
8	ARG	QD	9	TRP	HE1	4.76
9	TRP	HN	9	TRP	QB	3.55
9	TRP	HN	9	TRP	HD1	3.14
9	TRP	HA	9	TRP	HD1	3.27
9	TRP	HA	9	TRP	HE3	3.69
9	TRP	HA	10	ASN	HN	2.58
9	TRP	QB	10	ASN	HN	4.05
10	ASN	HN	10	ASN	HA	2.52
10	ASN	HN	10	ASN	HB2	3.35
10	ASN	HN	10	ASN	HB3	3.35

Table S6. Lowest energy pose DISCOVER refinement outputs.

**Complex**	**VdW**[a]	**Electr**[b]	**TOTAL**[c]
**FM635**/*h*MC3R	-75.7	-30.4	-106.1
**FM635**/*h*MC4R	-91.4	-45.4	-136.8

[a] Van der Waals energy; [b] Electrostatic energy; [c] Total energy; all terms are given in kcal/mol.

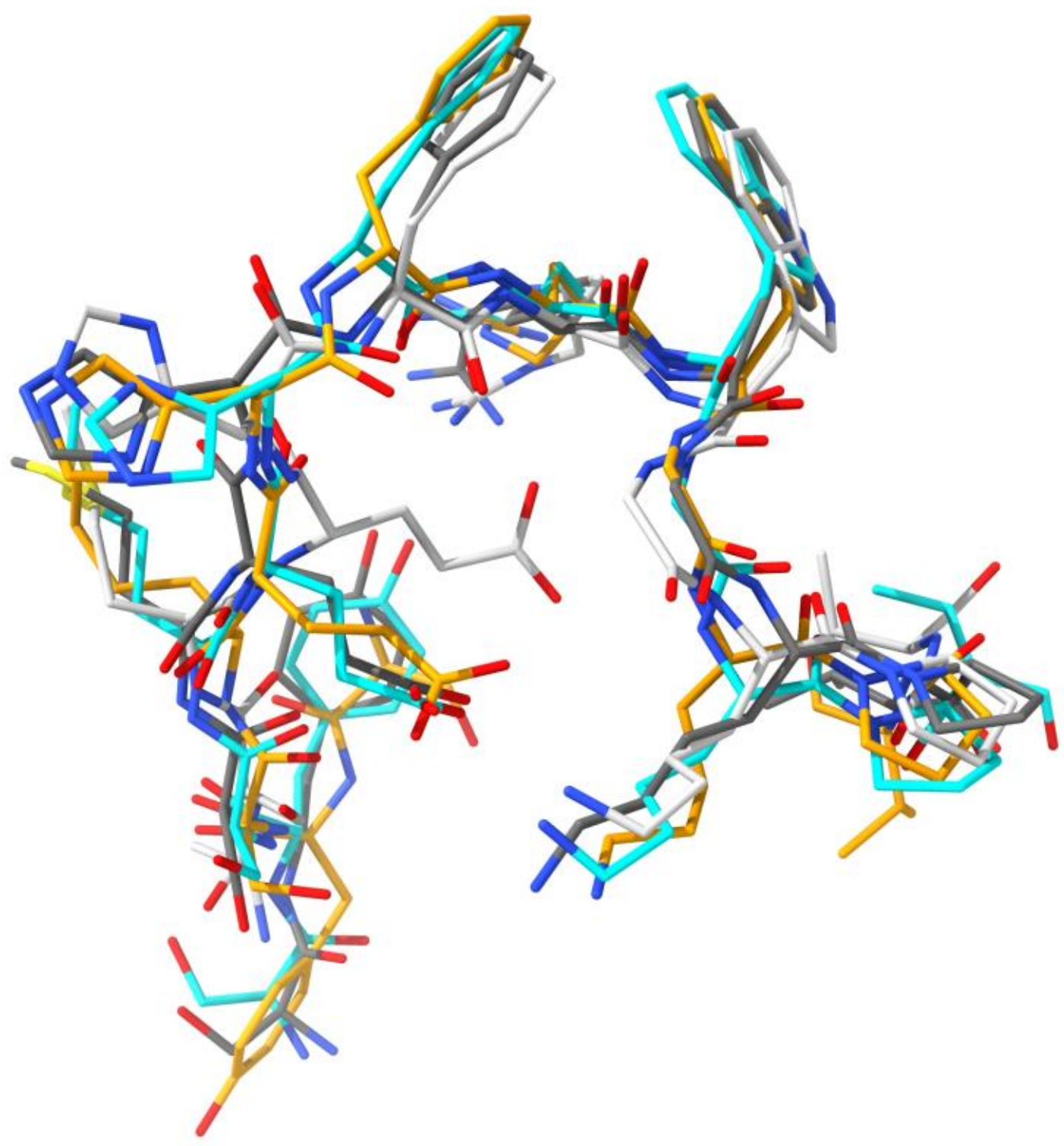

Figure S16. Structures of DPhe[7] containing peptides bound to various melanocortin receptors: α-MSH bound to MC1R (pdb code 7f4d, carbon atoms in light gray), MC3R (pdb code 8w8w, carbon atoms in gray), MC5R (pdb code 8inr, carbon atoms in cyan); NDP-MSH bound to MC4R (pdb code 7piv, carbon atoms in orange). Other heavy atoms (nitrogen, blue; oxygen, red).

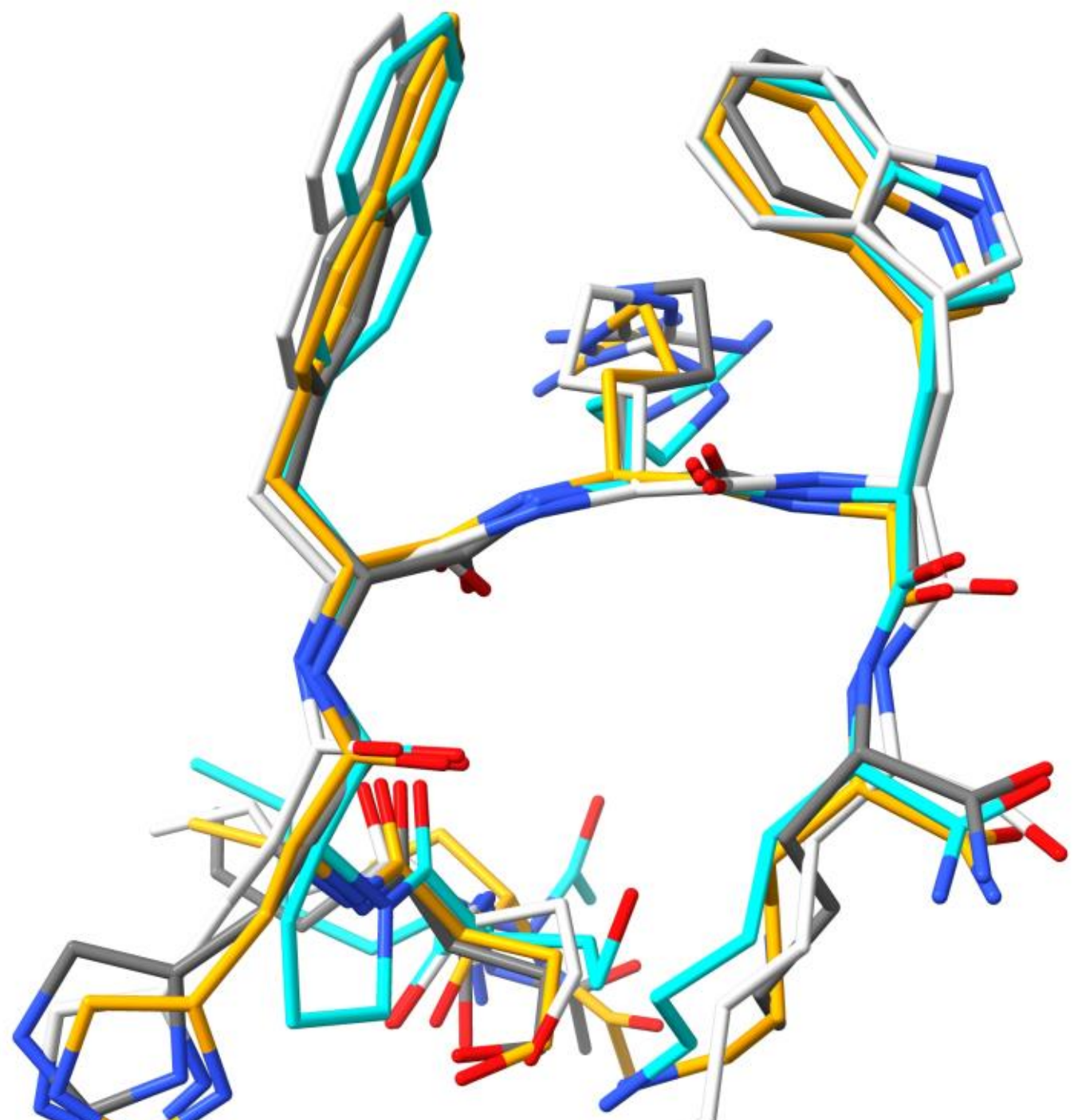

Figure S17. Structures of DNal[7] containing peptides bound to various melanocortin receptors: SHU-9119 bound to MC1R (pdb code 7f4i, carbon atoms in light gray), MC3R (pdb code 8kig, carbon atoms in gray), MC4R (pdb code 6w25, carbon atoms in orange); PG-901 bound to MC5R (pdb code 8iod, carbon atoms in cyan). Other heavy atoms (nitrogen, blue; oxygen, red).

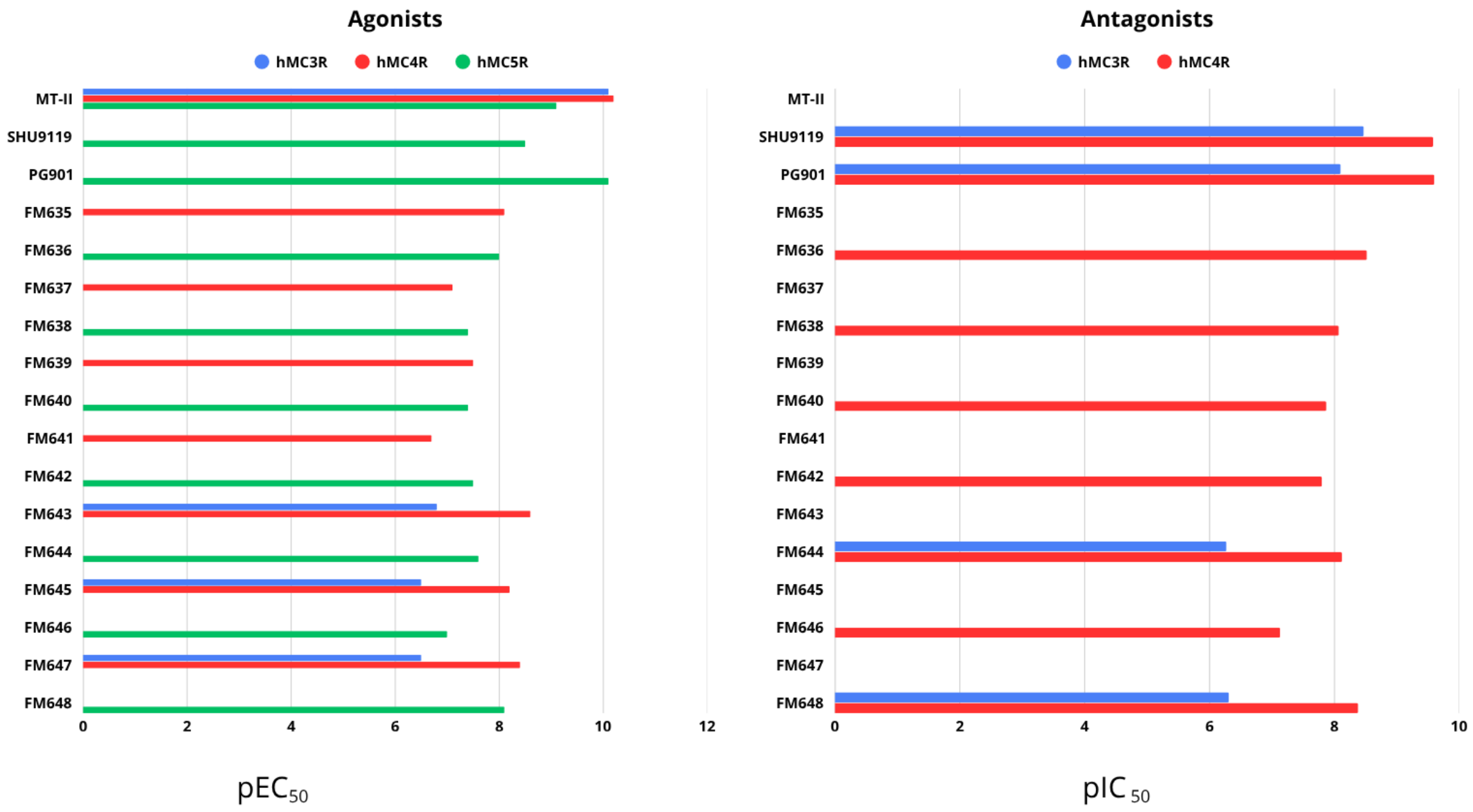

Figure S18. A bar chart illustrating the selectivity between agonist and antagonist activities of the synthesized compounds.
